# SENP6 Restrains NLRP3 Inflammasome Activation via DeSUMOylation-Driven K48-Linked Ubiquitination of NLRP3 in Acute Lung Injury

**DOI:** 10.34133/research.1069

**Published:** 2026-01-12

**Authors:** Angran Gu, Bailun Wang, Yi Zhang, Chang Sun, Yi Wei, Jie Yan, Yizheng Yang, Yichen Wang, Runmeng Liu, Ruirui Zhang, Jifa Liu, Changping Gu, Yuelan Wang

**Affiliations:** ^1^ Department of Anesthesiology, Shandong Provincial Hospital Affiliated to Shandong First Medical University, Jinan, Shandong 250021, China.; ^2^ School of Clinical and Basic Medical Sciences, Shandong First Medical University and Shandong Academy of Medical Sciences, Jinan, Shandong 250117, China.

## Abstract

The NLRP3 inflammasome is a pivotal component of the innate immune system, responding to infections and cellular damage. Its dysregulation has been implicated in numerous inflammatory diseases, although the mechanisms controlling its activation remain incompletely elucidated. Recent studies have highlighted the importance of posttranslational modifications, such as ubiquitination and SUMOylation, in regulating inflammasome activation. In this study, we demonstrate that SENP6, a SUMO-specific protease, negatively regulates NLRP3 inflammasome activation by promoting K48-linked polyubiquitination of NLRP3. SENP6-deficient macrophages exhibit enhanced NLRP3 activation and increased secretion of interleukin-1β (IL-1β) and IL-18, resulting in amplified inflammatory responses. Mechanistically, SENP6 interacts with NLRP3 and promotes its degradation through the autophagy–lysosomal pathway via K48-linked polyubiquitination. We further identified that SENP6 deSUMOylated NLRP3 at specific lysine residues (K23, K204, and K689), which was essential for maintaining NLRP3 stability. Additionally, SENP6 recruits the E3 ubiquitin ligase MARCHF7 to promote NLRP3 ubiquitination and subsequent degradation. In vivo, SENP6 deficiency exacerbates NLRP3 activation and lung inflammation in lipopolysaccharide-induced endotoxic shock-associated lung injury, and enhances inflammatory responses in alum-induced peritonitis. Our findings reveal a novel mechanism whereby SENP6 modulates NLRP3 inflammasome activation via SUMOylation, ubiquitination, and degradation, providing new insights into potential therapeutic strategies for inflammasome-related pathologies.

## Introduction

The NLRP3 inflammasome is a large multiprotein complex that, upon sensing priming signals mediated by pathogen-associated molecular patterns (PAMPs) and subsequent activation signals such as extracellular adenosine triphosphate (ATP), monosodium urate (MSU) crystals, nigericin, and mitochondrial reactive oxygen species (ROS), induces the activation of caspase-1 [1–3]. Activated caspase-1 then promotes the maturation and secretion of pro-inflammatory cytokines, including interleukin-1β (IL-1β) and interleukin-18 (IL-18) [4,5]. During this process, posttranslational modifications (PTMs), including ubiquitination, SUMOylation, UFMylation, ISGylation, and palmitoylation, play critical roles in regulating the assembly and activation of the NLRP3 inflammasome [6–10]. These modifications dynamically modulate the stability, subcellular localization, and activation state of NLRP3, thereby fine-tuning its function in immune responses [1].

The intracellular abundance of NLRP3 protein is considered one of the rate-limiting steps in inflammasome activation [11]. For instance, UFMylation of NLRP3 selectively inhibits K63-linked ubiquitination and the subsequent autophagic degradation, thereby maintaining its protein stability [8]. In addition, tripartite motif-containing protein 28 (TRIM28) promotes NLRP3 SUMOylation by inhibiting its K48-linked ubiquitination, thus preventing protein degradation [12].

SUMOylation, a dynamic and reversible PTM analogous to ubiquitination, is essential for regulating substrate protein stability, subcellular localization, activity, and protein–protein interactions [13]. This process involves the covalent attachment of SUMO proteins to lysine residues on target proteins, facilitated by E1 activating enzymes, E2 conjugating enzymes, and E3 ligases [14,15]. Conversely, sentrin/SUMO-specific proteases (SENPs) precisely remove SUMO modifications. To date, 6 members of the SENP family (SENP1 to SENP3 and SENP5 to SENP7) have been identified, all of which play crucial roles in maintaining SUMO homeostasis [16,17].

Studies have shown that SENP6 can preferentially SUMO2/3 chains from substrate proteins, exerting dual roles in regulating protein function, expression, cell cycle, localization, and stability [18,19]. For example, in neurons following ischemic stroke, SENP6 promotes neuronal apoptosis by deSUMOylating the transcription factor nuclear factor erythroid 2-related factor 2 [20]. In contrast, SENP6 maintains mitochondrial homeostasis and protects mitochondrial function by deSUMOylating TOM40 [21].

Furthermore, SENP6 participates in regulating the localization and nuclear aggregation of DNA damage response proteins [22]. Nevertheless, the precise mechanisms by which SENP6 regulates NLRP3 inflammasome activation and contributes to inflammation remain largely unknown.

Herein, we demonstrate that SENP6 interacts with NLRP3, preferentially removes SUMO2/3 chains at K23, K204, and K689 of NLRP3, and recruits MARCHF7 to increase K48-linked ubiquitination, leading to its autophagy-mediated degradation and reduced inflammasome activation. In vivo, SENP6-deficient mice exhibit markedly enhanced inflammatory responses in lipopolysaccharide (LPS)-induced endotoxic acute lung injury (ALI) and alum-induced peritonitis. Collectively, our findings identify SENP6 as a key regulator of NLRP3 inflammasome activity and suggest that targeting SENP6 may provide a promising therapeutic strategy for inflammatory diseases.

## Results

### Up-regulation of SENP6 during NLRP3 inflammasome activation

**Fig. 1.**
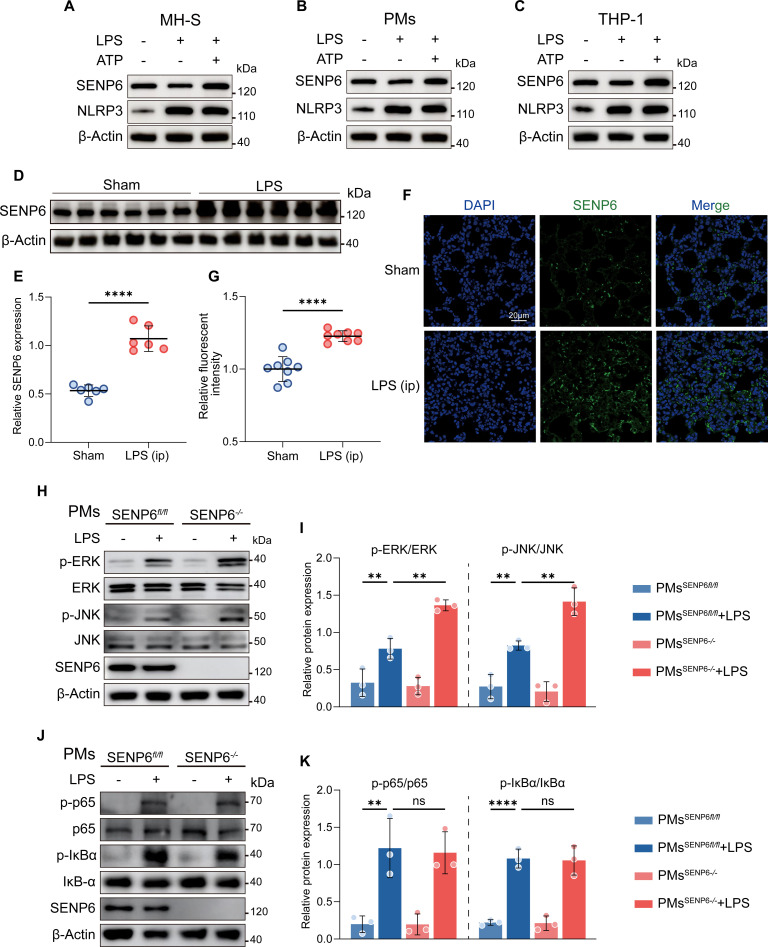
Up-regulation of SENP6 occurs during NLRP3 inflammasome activation. (A to C) Immunoblot analysis of SENP6 and NLRP3 in whole-cell lysates (WCLs) from MH-S (A), PMs (B), and THP-1 (C) cells primed with LPS (200 ng/ml, 4 h) and activated with ATP (2 mM, 45 min). (D and E) Western blot analysis and quantification of SENP6 protein levels in lung tissues from sham-treated mice or mice intraperitoneally (ip) injected with LPS (25 mg/kg, 12 h) (*n* = 6). (F and G) Immunofluorescence staining and quantitative analysis of SENP6 in lung sections from sham-treated or LPS-injected mice (*n* = 8). Scale bar, 20 μm. (H to K) Immunoblot analysis of phosphorylated and total ERK, JNK, p65, and IκBα in WCLs from LPS-stimulated SENP6*^fl/fl^* and SENP6*^fl/fl^ Lyz2-cre* PMs. Data are presented as mean ± SD. Statistical analysis was performed using Student’s *t* test (E and G) or two-way ANOVA with Bonferroni test (I and K), based on *n* = 3 independent biological experiments. *P < 0.05, **P < 0.01, ***P < 0.001, ****P < 0.0001.

To investigate the role of SENP6 in the regulation of NLRP3 inflammasome activation, we first assessed SENP6 expression in various macrophage types, including mouse alveolar macrophages (MH-S), mouse peritoneal macrophages (PMs), and THP-1-derived macrophages, upon stimulation with LPS and ATP. Our results consistently demonstrated that SENP6 expression was up-regulated in different types of macrophages (MH-S, PMs, and THP-1-derived macrophages) during NLRP3 inflammasome activation (Fig. 1A to C). These findings suggest that SENP6, as a crucial regulator of deSUMOylation, is closely related to NLRP3 inflammasome activation.

Next, we examined the in vivo dynamics of SENP6 expression during NLRP3 activation. In wild-type (WT) mice, we observed a marked up-regulation of SENP6 in the lungs following intraperitoneal injection of LPS. This increase in SENP6 expression was further confirmed by immunofluorescence staining (Fig. 1D to G), reinforcing the relevance of SENP6 in the context of NLRP3 inflammasome activation.

To explore the functional role of SENP6 in NLRP3 inflammasome activation, we employed RNA interference to silence SENP6 expression. A small interfering RNA (siRNA) targeting mouse SENP6 was transfected into MH-S cells, and knockdown efficiency was confirmed at both the mRNA and protein levels. Importantly, the silencing of SENP6 did not affect the expression of other members of the SENP family (Fig. S1A and B). Additionally, we designed a specific si*Senp6*-resistant plasmid, and its transfection successfully restored SENP6 expression, further confirming the specificity of SENP6 knockdown (Fig. S1D). During NLRP3 inflammasome activation, we observed that siRNA-mediated knockdown of SENP6 significantly enhanced the phosphorylation of extracellular signal-regulated kinase (ERK) and c-Jun N-terminal kinase (JNK) while exerting minimal effects on the activity of the LPS-activated nuclear factor κB (NF-κB) pathway (Fig. S1E and F). To further investigate, SENP6-floxed mice were crossed with Lyz2-cre mice to generate a myeloid cell-specific SENP6 knockout mice, from which primary PMs were isolated. In agreement with observations in MH-S cells, LPS stimulation of SENP6-deficient PMs led to increased ERK and JNK phosphorylation, with negligible changes in NF-κB pathway activity (Fig. 1H to K). To further explore the role of the mitogen-activated protein kinase (MAPK) signaling pathway in SENP6-mediated NLRP3 priming, we used ERK inhibitor (U0126) and JNK inhibitor (SP600125) in both PMs and MH-S cells. The results demonstrated that inhibition of ERK with U0126 and JNK with SP600125 effectively down-regulated p-ERK and p-JNK expression, respectively. Importantly, both inhibitors also significantly reduced the expression of NLRP3 (Fig. S1G to L).

Collectively, these findings indicate that the absence of SENP6 promotes the initiation of LPS-induced NLRP3 inflammasome activation through the up-regulation of the MAPK signaling pathway.

### SENP6 deficiency promotes NLRP3 inflammasome activation

**Fig. 2.**
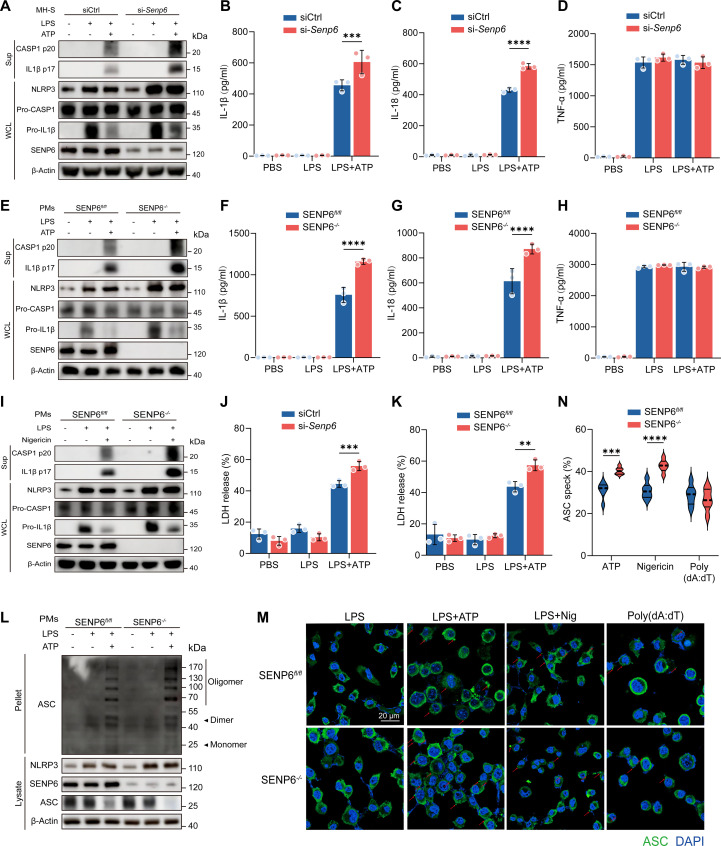
SENP6 deficiency promotes NLRP3 inflammasome activation. (A) Immunoblot analysis of proteins in supernatants (SNs) and WCL from SENP6*^fl/fl^* and SENP6*^fl/fl^ Lyz2-cre* PMs primed with LPS (200 ng/ml, 4 h) and activated with ATP (2 mM, 45 min). (B to D) Secretion of IL-1β (B), IL-18 (C), and TNF-α (D) in SN from LPS-primed and ATP-activated SENP6*^fl/fl^* and SENP6*^fl/fl^ Lyz2-cre* PMs (*n* = 3). (E) Immunoblot analysis of SN and WCL from MH-S cells transfected with si-Ctrl or si-*Senp6*, followed by LPS priming and ATP activation. (F to H) Secretion of IL-1β (F), IL-18 (G), and TNF-α (H) in SN from LPS-primed and ATP-activated MH-S cells transfected with si-Ctrl or si-*Senp6* (*n* = 3). (I) Immunoblot analysis of proteins in SN and WCL from SENP6*^fl/fl^* and SENP6*^fl/fl^ Lyz2-cre* PMs primed with LPS and activated with nigericin (10 μM, 1 h). (J and K) LDH release in SN from LPS-primed and ATP-activated SENP6*^fl/fl^* and SENP6*^fl/fl^ Lyz2-cre* PMs (*n* = 3). (L) Immunoblot analysis of ASC oligomerization in pellets and WCL from LPS-primed and ATP-activated SENP6*^fl/fl^* and SENP6*^fl/fl^ Lyz2-cre* PMs. (M) Representative confocal images of ASC specks in SENP6*^fl/fl^* and SENP6*^fl/fl^ Lyz2-cre* PMs after the indicated stimulations. ASC, green; nuclei, blue; white arrows indicate ASC specks. Scale bar, 10 μm. (N) Quantification of ASC speck numbers (*n* = 8). Data are presented as mean ± SD. Statistical analysis was performed using two-way ANOVA with Bonferroni test based on *n* = 3 independent biological experiments. *P < 0.05, **P < 0.01, ***P < 0.001, ****P < 0.0001.

Next, we evaluated whether SENP6 deficiency affects the activation of the NLRP3 inflammasome. In LPS-primed MH-S cells, stimulation with the NLRP3 activators ATP or nigericin significantly enhanced CASP-1 cleavage and promoted the release of IL-1β and IL-18 upon SENP6 knockdown, without affecting tumor necrosis factor-α (TNF-α) secretion (Fig. 2A to D and Fig. S2A to D). Consistently, SENP6-deficient PMs exhibited markedly increased ATP-induced CASP-1 cleavage and IL-1β and IL-18 release, with no significant change in TNF-α production (Fig. 2E to H). SENP6 deficiency also augmented nigericin-induced CASP-1 activation and cytokine release (Fig. 2I and Fig. S2F and G). In contrast, the absence of SENP6 had no detectable effect on AIM2 inflammasome activation by poly(deoxyadenylic acid–deoxythymidylic acid)[poly(dA:dT)] or the associated IL-1β and IL-18 release (Fig. S2E to G). Immunofluorescence staining revealed that SENP6 depletion substantially up-regulated NLRP3 and IL-1β protein levels in MH-S macrophages (Fig. S3). Given that NLRP3 inflammasome activation induces pyroptosis, we assessed lactate dehydrogenase (LDH) release as an indicator of cell death. SENP6-deficient macrophages showed significantly higher LDH release following LPS stimulation combined with ATP or nigericin (Fig. 2J and K and Fig. S2I and J). Moreover, NLRP3 inflammasome activation promotes apoptosis-associated speck-like protein containing a CARD (ASC) oligomerization and the assembly of ASC specks through pyrin domain interactions. Loss of SENP6 markedly increased both ASC oligomerization and ASC speck formation (Fig. 2L to N and Fig. S2H).

Collectively, these findings demonstrate that SENP6 deficiency selectively and robustly amplifies NLRP3 inflammasome activation.

### SENP6 interacts with NLRP3

**Fig. 3.**
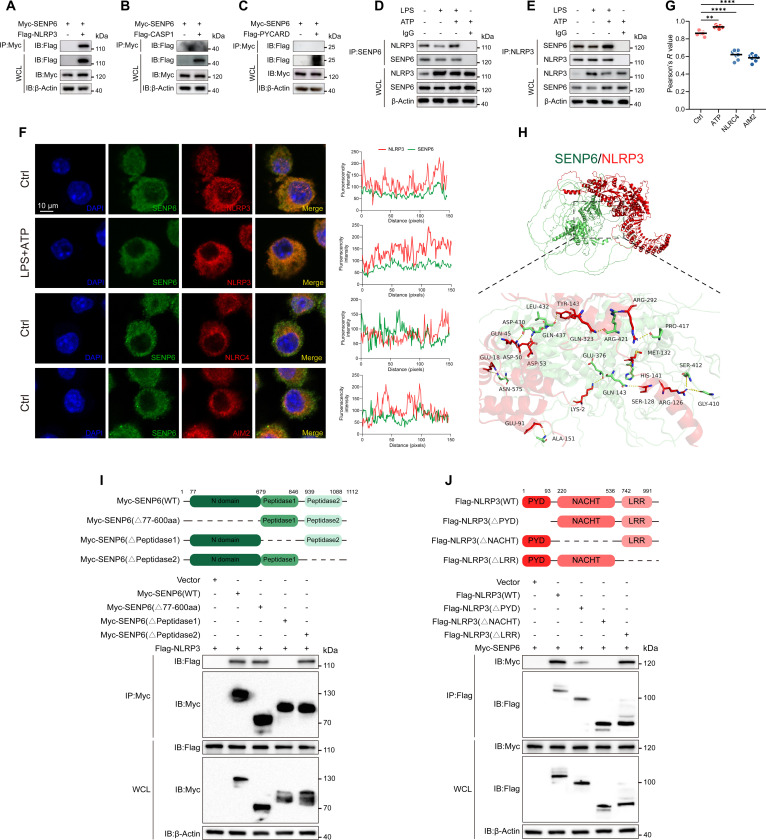
SENP6 interacts with NLRP3. (A to C) Immunoprecipitation (IP) and immunoblot analyses of lysates from HEK293T cells transfected with Myc-SENP6 and either Flag-NLRP3 (A), Flag-caspase-1 (B), or Flag-PYCARD (C). IP was performed using an anti-Myc antibody, followed by detection with anti-Flag. (D and E) IP analysis of endogenous SENP6–NLRP3 interactions in PMs stimulated with LPS alone or with LPS followed by ATP activation. (F) PMs were stimulated with LPS (200 ng/ml, 4 h) and ATP (2 mM, 45 min) in the LPS + ATP group. Confocal microscopy was employed to assess SENP6–NLRP3 colocalization and SENP6 interactions with NLRC4 or AIM2. SENP6 is shown in green; NLRP3, NLRC4, or AIM2 in red; and nuclei in blue (DAPI). Scale bar, 10 μm. Protein fluorescence intensity was quantified using ImageJ line-scan analysis. Images shown are representative of 3 independent experiments. (G) Pearson’s correlation coefficient were quantified using ImageJ software. (H) Protein–protein docking and schematic modeling of the predicted SENP6–NLRP3 interaction, generated using the GRAMM server (https://gramm.compbio.ku.edu/). Human NLRP3 is depicted in red and SENP6 in green. (I) Schematic representation of full-length SENP6 and its truncation mutants (upper panel). HEK293T cells were transfected with Myc-SENP6 or its mutants together with Flag-NLRP3. Lysates were immunoprecipitated with anti-Myc and analyzed by immunoblotting with the indicated antibodies. (J) Schematic representation of full-length NLRP3 and its truncation mutants (upper panel). HEK293T cells were transfected with Flag-NLRP3 or its mutants together with Myc-SENP6. Lysates were immunoprecipitated with anti-Flag and analyzed by immunoblotting with the indicated antibodies. Data are presented as mean ± SD. Statistical analysis was performed using one-way ANOVA with Bonferroni test (*n* = 8). **P < 0.01, ****P < 0.0001.

To further elucidate the mechanism by which SENP6 regulates NLRP3 activation, we expressed Myc-tagged SENP6 and Flag-tagged NLRP3, caspase-1, and ASC in HEK293T cells and conducted co-immunoprecipitation (CO-IP) assays. The in vitro CO-IP results revealed that SENP6 specifically interacted with NLRP3 (Fig. 3A), but did not bind to caspase-1 or ASC (Fig. 3B and C). In line with these findings, endogenous SENP6 was also shown to interact with NLRP3 in MH-S cells. Furthermore, treatment with the NLRP3 inflammasome activator ATP significantly enhanced the interaction between SENP6 and NLRP3 (Fig. 3D and E). Consistent with these results, immunofluorescence staining demonstrated the colocalization of SENP6 with NLRP3, and importantly, the colocalization of SENP6 with NLRP3 was significantly enhanced after LPS and subsequent ATP stimulation. No colocalization was observed with AIM2 or NLRC4 (Fig. 3F and G).

Then, to further investigate the specific interaction surface between SENP6 and NLRP3, we utilized the GRAMM server for structural predictions [23]. The computational analysis revealed a close interaction between SENP6 and NLRP3 (Fig. 3H), which is in agreement with our experimental data.

Next, to identify the structural domains responsible for the interaction between SENP6 and NLRP3, we constructed 3 Myc-tagged SENP6 truncation mutants, each representing one of its domains: the N domain, Peptidase1, and Peptidase2 [18]. CO-IP experiments revealed that only the SENP6 mutant lacking the Peptidase1 domain failed to interact with NLRP3, whereas mutants lacking the N domain or Peptidase2 domain still retained the ability to bind NLRP3 (Fig. 3I). These results suggest that the Peptidase1 domain of SENP6 is crucial for its interaction with NLRP3.

NLRP3 is composed of a C-terminal leucine-rich repeat (LRR) domain, a central NACHT domain, and an N-terminal pyrin (PYD) domain [2]. To pinpoint the specific regions involved in this interaction, we constructed corresponding truncation mutants of NLRP3 and performed CO-IP experiments. We found that deletion of the PYD domain in NLRP3 impaired its interaction with SENP6, while loss of the NACHT domain completely abrogated the binding. In contrast, removal of the LRR domain had no evident effect on this interaction (Fig. 3J). These findings collectively demonstrate that SENP6 interacts with NLRP3 through its Peptidase1 domain and specifically binds to the PYD and NACHT domains of NLRP3.

### SENP6 promotes autophagic degradation of NLRP3

**Fig. 4.**
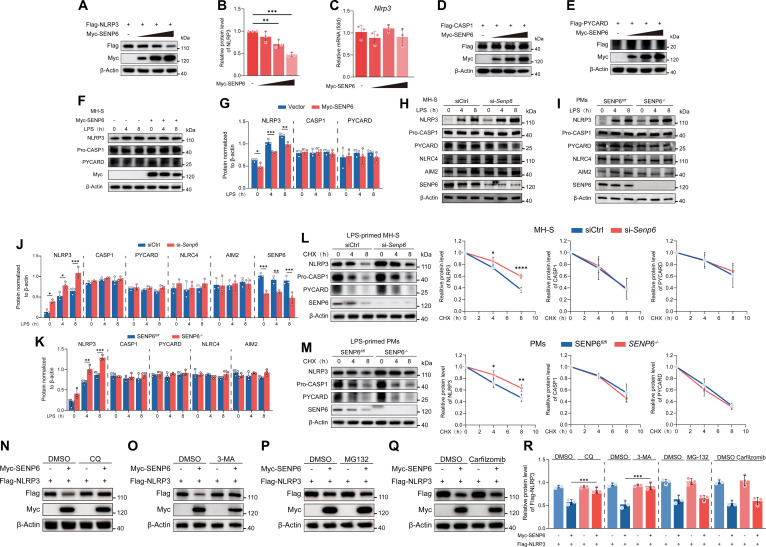
SENP6 promotes autophagic degradation of NLRP3. (A to C) HEK293T cells were transfected with Flag-NLRP3 and increasing amounts of Myc-SENP6 for 24 h. Lysates were analyzed by immunoblotting (A), with densitometric quantification of NLRP3 protein levels (B). NLRP3 mRNA expression was measured by RT-qPCR (C) (*n* = 3). (D and E) HEK293T cells were transfected with Flag-CASP1 or Flag-PYCARD together with increasing amounts of Myc-SENP6. Lysates were subjected to immunoblotting to assess CASP1 and PYCARD expression. (F and G) MH-S cells transfected with Myc-SENP6 were stimulated with LPS (200 ng/ml) for 0, 4, or 8 h. Lysates were collected for immunoblotting (F), followed by densitometric analysis to quantify protein expression (G) (*n* = 3). (H to K) SENP6-knockdown MH-S cells and SENP6-deficient PMs cells were stimulated with LPS for the indicated times. Lysates were analyzed by immunoblotting (H and I) and densitometric quantification (J and K) (*n* = 3). (L and M) MH-S and SENP6-knockdown MH-S cells were treated with LPS (200 ng/ml) for 4 h, followed by CHX (100 μg/ml) for the indicated times. Lysates were analyzed by immunoblotting (L), and NLRP3 protein levels were quantified (M) (*n* = 3). (N to R) HEK293T cells transfected with Flag-NLRP3 and Myc-SENP6 were treated for 6 h with DMSO (vehicle), chloroquine (CQ; 50 μM) (N), 3-methyladenine (3-MA, 10 mM) (O), MG132 (10 μM) (P), or carfilzomib (100 nM) (Q) followed by immunoblot analysis. Quantitative statistical analysis of Flag-NLRP3 expression levels in each group was performed (R). Data are presented as mean ± SD. Statistical analysis was performed using one-way ANOVA (B and C) or two-way ANOVA with Bonferroni test based on *n* = 3 independent biological experiments. *P < 0.05, **P < 0.01, ***P < 0.001, ****P < 0.0001.

Given the negative regulatory role of SENP6 in NLRP3 inflammasome activation and its direct interaction with NLRP3, we hypothesized that SENP6 may regulate NLRP3 protein expression. Experimental data revealed that overexpression of SENP6 significantly reduced NLRP3 protein levels (Fig. 4A and B), without affecting NLRP3 mRNA abundance (Fig. 4C). Additionally, the protein levels of CASP1 and PYCARD were not altered (Fig. 4D and E). Similarly, in MH-S cells, overexpression of SENP6 led to a notable decrease in endogenous NLRP3 expression, while the protein expression of CASP1 and PYCARD remained unaffected (Fig. 4F and G). Conversely, in SENP6-knockdown MH-S cells and SENP6-deficient PMs, NLRP3 protein levels were significantly elevated, with no change in CASP1 and PYCARD protein expression, nor in the levels of the noncanonical inflammasomes AIM2 and NLRC4 (Fig. 4H to K).

To further investigate the mechanism by which SENP6 regulates NLRP3 protein levels, we performed cycloheximide (CHX) chase experiments. We observed that knockdown or deletion of SENP6 delayed NLRP3 degradation, while the degradation of CASP1, PYCARD, AIM2, and NLRC4 remained unchanged (Fig. 4L and M). This suggests that SENP6 modulates NLRP3 protein levels by enhancing its degradation.

Protein degradation in eukaryotic cells is primarily regulated by 2 systems: the ubiquitin–proteasome system and the autophagy–lysosome system [24]. To identify which degradation pathway is involved in SENP6-mediated NLRP3 degradation, we utilized specific inhibitors. We found that the lysosomal inhibitor chloroquine (CQ) and the autophagy inhibitor 3-methyladenine (3-MA) significantly suppressed SENP6-induced NLRP3 degradation, while the proteasome inhibitor MG132 and carfilzomib exerted no effect (Fig. 4N to R). These results indicate that SENP6 predominantly facilitates NLRP3 degradation via the autophagy–lysosome pathway.

### SENP6 enhances K48-linked ubiquitination of NLRP3

**Fig. 5.**
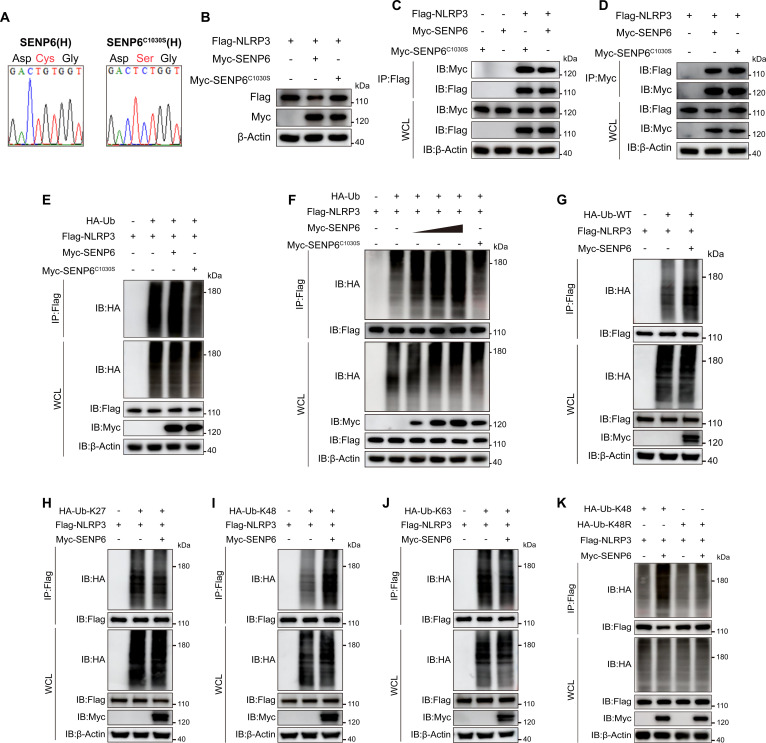
SENP6 enhances K48-linked ubiquitination of NLRP3. (A) DNA sequencing verification of human Myc-SENP6 and the catalytically inactive mutant Myc-SENP6^C1030S^. (B) Immunoblot of HEK293T lysates transfected with Flag-NLRP3 and either Myc-SENP6 or Myc-SENP6^C1030S^. (C and D) HEK293T cells transfected with Flag-NLRP3 and either Myc-SENP6 or Myc-SENP6^C1030S^ were immunoprecipitated with anti-Flag (C) or anti-Myc (D) antibodies, followed by immunoblotting. (E) Lysates from HEK293T cells coexpressing HA-tagged ubiquitin (HA-Ub), Flag-NLRP3, and either Myc-SENP6 or Myc-SENP6^C1030S^ were immunoprecipitated with anti-Flag and probed with anti-HA to detect ubiquitination. (F) HEK293T cells were cotransfected with HA-Ub, Flag-NLRP3, and increasing amounts of Myc-SENP6 and Myc-SENP6^C1030S^. Lysates were immunoprecipitated with anti-Flag and probed with anti-HA. (G to J) HEK293T cells expressing HA-tagged WT, K27-, K48-, or K63-linked ubiquitin, together with Flag-NLRP3 and Myc-SENP6, were immunoprecipitated with anti-Flag and analyzed by anti-HA immunoblotting. (K) Lysates from HEK293T cells coexpressing Flag-NLRP3, Myc-SENP6, and either HA-Ub-K48 or HA-Ub-K48R were immunoprecipitated with anti-Flag and probed with anti-HA.

Ubiquitination of the NLRP3 inflammasome plays a crucial role in regulating its function [25]. To further investigate the role of SENP6 in NLRP3 ubiquitination, we explored whether SENP6 modulates NLRP3 ubiquitination through its capacity to reduce NLRP3 SUMOylation. Initially, we assessed whether SENP6-mediated NLRP3 degradation is dependent on its enzymatic activity. It has been previously reported that the cysteine residue at position 1030 of SENP6 is essential for its catalytic function [19]. Accordingly, we substituted this cysteine with serine to generate the SENP6-C1030S mutant (Fig. 5A). As anticipated, the SENP6-C1030S mutant lost its ability to induce NLRP3 degradation (Fig. 5B). However, this mutant did not disrupt the interaction between SENP6 and NLRP3 (Fig. 5C and D), suggesting that SENP6 suppresses NLRP3 expression through its enzymatic activity.

Subsequently, we examined whether SENP6 could regulate NLRP3 ubiquitination. We found that overexpression of SENP6 significantly enhanced the polyubiquitination of NLRP3 in a dose-dependent manner. Notably, the enzymatically inactive SENP6 mutant also failed to mediate NLRP3 ubiquitination (Fig. 5E and F). Given that NLRP3 can undergo various types of ubiquitination, each of which may serve distinct biological functions, we next sought to determine which specific type of ubiquitin linkage on NLRP3 is regulated by SENP6. To do so, we generated K27, K48, and K63 ubiquitin mutants, each with a single lysine residue available for linkage. Remarkably, overexpression of SENP6 significantly increased NLRP3 ubiquitination in both WT and K48 ubiquitin-transfected cells, while no significant changes were observed in K27 or K63 ubiquitin-transfected cells (Fig. 5G to J).

Further, we constructed the K48R mutant, in which all lysine residues were replaced with arginine. We observed that the K48R mutation abolished SENP6’s ability to enhance NLRP3 ubiquitination (Fig. 5K). Collectively, these findings indicate that SENP6 promotes NLRP3 inflammasome activation by facilitating K48-linked polyubiquitination.

### SENP6 recruits MARCHF7 to promote the K48-linked ubiquitination of NLRP3

SENP6 is traditionally recognized for its deSUMOylation activity but is not thought to possess ubiquitin ligase activity. However, given that SENP6 promotes the polyubiquitination of NLRP3, we hypothesized that SENP6 might regulate NLRP3 ubiquitination by recruiting an E3 ubiquitin ligase. Several E3 ligases, such as TRIM31 [26], Pellino2 [27], FBXL2 [28], gp78 [29], and MARCHF7 [30], have been shown to participate in the polyubiquitination and degradation of NLRP3. Among these, MARCHF7 attracted our attention due to its ability to promote K48-linked polyubiquitination of NLRP3, thereby accelerating its degradation through the autophagy–lysosomal pathway. Interestingly, our previous studies have demonstrated that SENP6 mediates K48-linked ubiquitination of NLRP3 and its subsequent autophagic degradation. Based on these findings, we speculated that SENP6 regulates NLRP3 ubiquitination by recruiting MARCHF7.

**Fig. 6.**
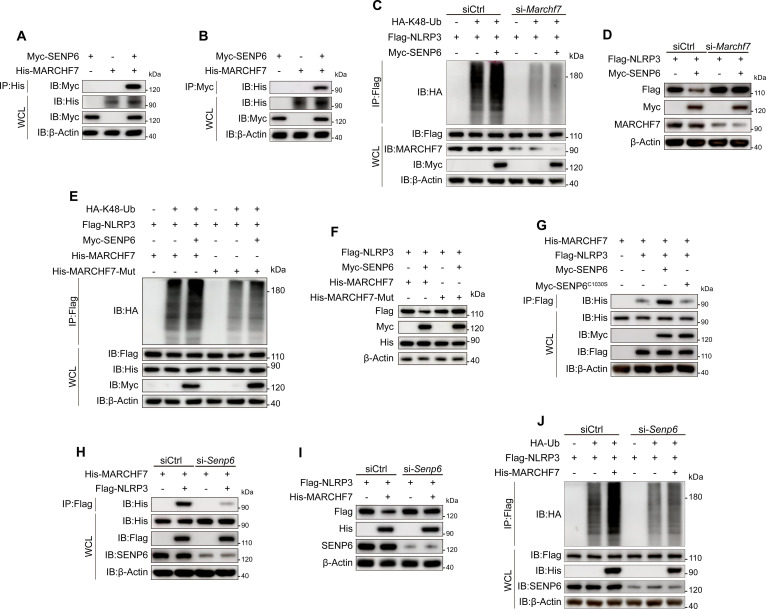
SENP6 recruits MARCHF7 to promote the K48-linked ubiquitination of NLRP3. (A and B) HEK293T cells cotransfected with His-MARCHF7 and Myc-SENP6 were lysed for IP and immunoblotting. (C) HA-K48-Ub, Flag-NLRP3, and Myc-SENP6 were cotransfected into control or *Marchf7*-knockdown HEK293T cells, and lysates were immunoblotted and immunoprecipitated with anti-Flag. (D) HEK293T cells were transfected with Flag-NLRP3 plasmid in the presence or absence of Myc-SENP6, together with either si-Ctrl or si-*Marchf7*, and cultured for 48 h. WCLs were then analyzed by immunoblotting. (E) HEK293T cells were cotransfected with HA-K48-Ub, Flag-NLRP3, Myc-SENP6, and His-MARCHF7 or His-MARCHF7 (W589A/I556A). Lysates were then immunoblotted and immunoprecipitated with anti-HA antibodies. (F) HEK293T cells were cotransfected with Flag-NLRP3, Myc-SENP6, and His-MARCHF7 or His-MARCHF7 (W589A/I556A). WCLs were subsequently analyzed by immunoblotting. (G) HEK293T cells were cotransfected with Flag-NLRP3, His-MARCHF7, Myc-SENP6, or Myc-SENP6^C1030S^, treated with CQ (10 μM, 6 h), and subjected to CO-IP followed by immunoblotting to assess the His-MARCHF7–Flag-NLRP3 interaction. (H) His-MARCHF7 and Flag-NLRP3 were cotransfected into control or *Senp6*-knockdown HEK293T cells and analyzed for their interaction by IP and immunoblotting. (I) His-MARCHF7 and Flag-NLRP3 were cotransfected into control or *Senp6*-knockdown HEK293T cells for immunoblotting. (J) HA-K48-Ub, Flag-NLRP3, and His-MARCHF7 were cotransfected into control or *Senp6*-knockdown HEK293T cells, and lysates were immunoblotted and immunoprecipitated with anti-Flag.

To test this hypothesis, we first confirmed the interaction between SENP6 and MARCHF7 through CO-IP assays (Fig. 6A and B). Further investigation revealed that knockdown of *Marchf7* completely abolished SENP6-mediated K48-linked NLRP3 ubiquitination and its degradation (Fig. 6C and D), indicating that SENP6’s effect on NLRP3 ubiquitination is dependent on MARCHF7. Then, we transfected cells with a si*Marchf7*-resistant plasmid, which significantly restored MARCHF7 expression following its knockdown. Notably, the diminished ability of SENP6 to mediate NLRP3 ubiquitination and degradation caused by MARCHF7 knockdown was completely restored, confirming the specificity of MARCHF7 in the SENP6-mediated regulation of NLRP3 (Fig. S4C and D).

To further explore the functional role of MARCHF7 in this process, we constructed MARCHF7 W589A/I556A mutants to inhibit its enzymatic activity. Results showed that after MARCHF7 enzymatic activity was lost, SENP6’s ability to promote K48-linked ubiquitination of NLRP3 was significantly reduced. Furthermore, SENP6’s capacity to facilitate NLRP3 degradation was also markedly diminished. These results highlight the critical role of MARCHF7 enzymatic activity in SENP6-mediated regulation of NLRP3 (Fig. 6E and F).

We next observed that overexpression of SENP6 in HEK293T cells enhanced the interaction between MARCHF7 and NLRP3. Notably, the enzymatically inactive SENP6-C1030S mutant weakened the interaction between MARCHF7 and NLRP3 (Fig. 6G). In contrast, knockdown of SENP6 in HEK293T cells impaired the binding between MARCHF7 and NLRP3 (Fig. 6H). These results suggest that the interaction between MARCHF7 and NLRP3 is dependent on SENP6.

Finally, we explored whether SENP6 is essential for MARCHF7-mediated NLRP3 polyubiquitination and degradation. The results showed that knockdown of SENP6 reversed the decrease in NLRP3 protein levels induced by MARCHF7 overexpression (Fig. 6I). Moreover, transfection of SENP6 siRNA significantly reduced MARCHF7-induced NLRP3 polyubiquitination (Fig. 6J). Collectively, these data demonstrate that SENP6 promotes K48-linked polyubiquitination and degradation of NLRP3 by recruiting MARCHF7.

### SENP6 mediates deSUMOylation of NLRP3 at K23, K204, and K689

**Fig. 7.**
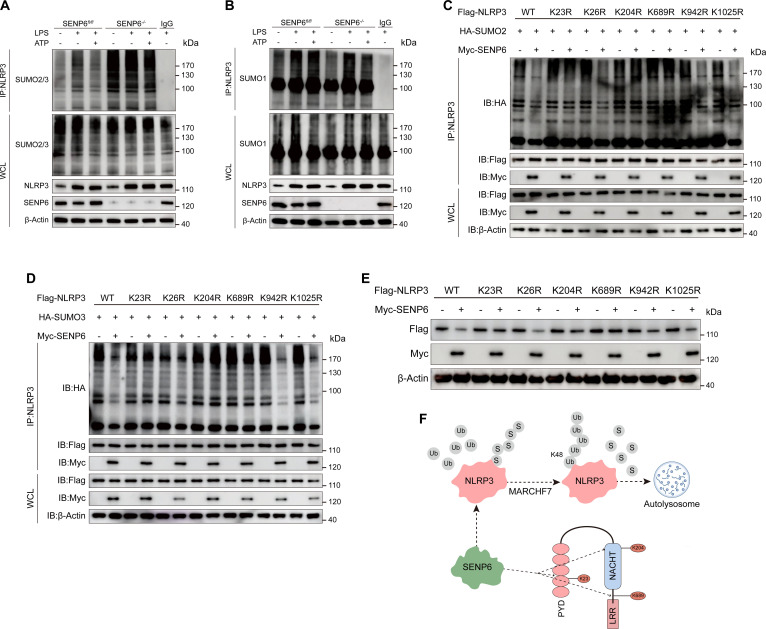
SENP6 mediates deSUMOylation of NLRP3 at K23, K204, and K689. (A and B) Immunoblot analysis of PM lysates from SENP6*^fl/fl^* and SENP6*^fl/fl^ Lyz2-cre* mice. Cells were stimulated with LPS for 4 h and subsequently stimulated with ATP for 1 h. SUMOylation levels of NLRP3 (SUMO2/3 and SUMO1) were detected using an anti-NLRP3 antibody. (C and D) HEK293T cells were cotransfected with Myc-SENP6, HA-SUMO2 (C) or HA-SUMO3 (D), and Flag-tagged WT or mutant NLRP3 constructs. Cell lysates were immunoprecipitated using an anti-HA antibody, followed by immunoblot analysis to assess NLRP3 SUMOylation. (E) HEK293T cells were cotransfected with Myc-SENP6 and either WT or mutant Flag-NLRP3. Immunoblot analysis was performed on WCL. (F) Schematic representation illustrating that SENP6 removes SUMO2/3 modifications from NLRP3 at lysine residues K23, K204, and K689, enhances K48-linked polyubiquitination, and promotes its degradation via the autophagy–lysosomal pathway.

Since SENP6 is a pivotal deSUMOylating enzyme, we sought to further elucidate the specific mechanism by which SENP6 deSUMOylates NLRP3. Prior research has demonstrated that SENP6 preferentially cleaves SUMO2/3 chains. To verify this specificity, we assessed the levels of SUMO2/3 and SUMO1 modifications on NLRP3 in PMs deficient in SENP6. Our results revealed that SENP6 knockout led to a significant accumulation of SUMO2/3-modified NLRP3, while SUMO1 modification levels showed minimal change (Fig. 7A and B). These findings provide compelling evidence for the specific role of SENP6 in the deSUMOylation of NLRP3, highlighting its selective action on SUMO2/3 chains.

To identify the precise SUMOylation sites on NLRP3, we utilized the SumoPlot analysis tool (Sumo.biocuckoo.cn), which predicted 6 lysine residues (K23, K26, K204, K689, K942, and K1025) as potential targets for SUMOylation. These residues were individually mutated to arginine, resulting in K23R, K26R, K204R, K689R, K942R, and K1025R NLRP3 mutants for further SUMOylation analysis. Our findings demonstrated that in HEK293T cells cotransfected with K23R, K204R, and K689R mutants along with the SUMO2 plasmid, the deSUMOylation effect of SENP6 on NLRP3 was abolished (Fig. 7C). In contrast, no such effect was observed in cells transfected with other mutant constructs. Consistent results were obtained when SUMO3 plasmids were cotransfected (Fig. 7D).

Furthermore, additional experiments revealed that in cells cotransfected with the K23R, K204R, and K689R mutants, SENP6 was unable to promote NLRP3 degradation, suggesting that these specific lysine residues are critical for the functional interaction between SENP6 and NLRP3 (Fig. 7E). Taken together, these findings provide robust evidence that SENP6 regulates NLRP3 expression by deSUMOylating NLRP3 at the K23, K204, and K689 residues, highlighting the critical role of these sites in the modulation of NLRP3 stability.

### SENP6 deficiency promotes NLRP3-dependent inflammation

**Fig. 8.**
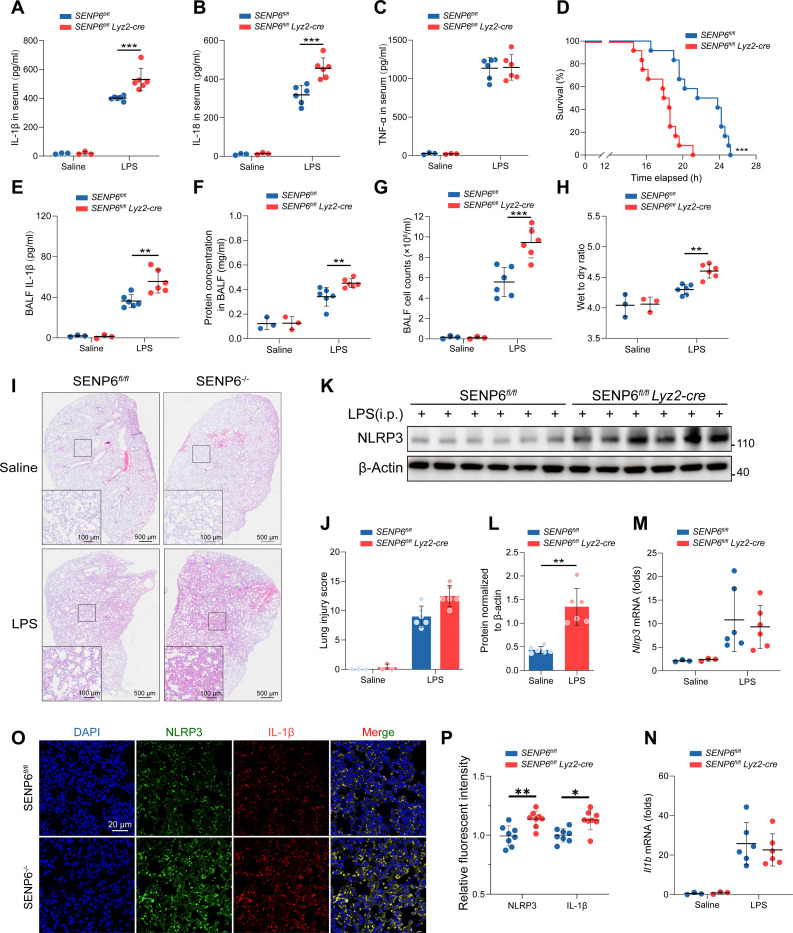
SENP6 deficiency promotes NLRP3-dependent inflammation. (A to C) SENP6*^fl/fl^* and SENP6*^fl/fl^ Lyz2-cre* mice (saline: *n* = 3; LPS: *n* = 6) were intraperitoneally injected with either saline (100 μl) or LPS (25 mg/kg) for 12 h. Serum levels of IL-1β (A), IL-18 (B), and TNF-α (C) were measured by ELISA. (D) Survival rates of SENP6*^fl/fl^* and SENP6*^fl/fl^ Lyz2-cre* mice following intraperitoneal injection of LPS (25 mg/kg) (*n* = 12). (E) ELISA analysis of IL-1β levels in bronchoalveolar lavage fluid (BALF) from SENP6*^fl/fl^* and SENP6*^fl/fl^ Lyz2-cre* mice 12 h after intraperitoneal LPS injection (saline: *n* = 3; LPS: *n* = 6). (F and G) BALF protein concentration (F) and total cell counts (G) in SENP6*^fl/fl^* and SENP6*^fl/fl^ Lyz2-cre* mice 12 h after intraperitoneal LPS injection (saline: *n* = 3; LPS: *n* = 6). (H) Lung wet-to-dry (W/D) weight ratio in SENP6*^fl/fl^* and SENP6*^fl/fl^ Lyz2-cre* mice after 12 h of intraperitoneal LPS injection (saline: *n* = 3; LPS: *n* = 6). (I and J) Representative images of H&E-stained lung sections from SENP6*^fl/fl^* and SENP6*^fl/fl^ Lyz2-cre* mice. Scale bars, 500 μm for whole-lung sections, and 100 μm for zoomed-in images (saline: *n* = 3; LPS: *n* = 6). (K and L) Immunoblot analysis of lung lysates from SENP6*^fl/fl^* and SENP6*^fl/fl^ Lyz2-cre* mice 12 h after LPS injection (*n* = 6). (L) NLRP3 expression levels were quantified using ImageJ software and normalized to β-actin. (M and N) RT-PCR analysis of *Nlrp3* (M) and *Il1b* (N) mRNA expression in the lungs of SENP6*^fl/fl^* and SENP6*^fl/fl^ Lyz2-cre* mice 12 h after intraperitoneal LPS injection (saline: *n* = 3; LPS: *n* = 6). (O) Representative immunofluorescence images showing NLRP3 (green) and IL-1β (red) expression in lung tissues from both groups 12 h after intraperitoneal LPS injection. Nuclei were counterstained with DAPI (blue) (*n* = 6). Scale bar, 20 μm. (P) Quantification of fluorescence intensity was performed using ImageJ (*n* = 8). Data are presented as mean ± SD. Statistical analysis was performed using two-way ANOVA with Bonferroni test or Student’s *t* test (L), based on *n* = 3 independent biological replicates for the saline group and *n* = 6 independent biological replicates for the LPS group. *P < 0.05,**P < 0.01, and ***P < 0.001.

Finally, we employed an LPS-induced endotoxic shock mouse model to investigate the regulatory role of SENP6 in NLRP3 inflammasome activation in vivo. We found that, compared to SENP6*^fl/fl^* mice, SENP6-deficient mice exhibited significantly increased levels of IL-1β and IL-18 in serum, while TNF-α production showed no difference (Fig. 8A to C), and their survival rate was also markedly reduced (Fig. 8D). Furthermore, compared with SENP6*^fl/fl^* mice, SENP6*^fl/fl^ Lyz2-cre* mice exhibited significantly increased secretion of IL-1β (Fig. 8E) and IL-18 in bronchoalveolar lavage fluid (BALF) following LPS stimulation, while TNF-α levels remained unchanged (Fig. S5A and B). In addition, both total protein concentration and cell counts in BALF were markedly elevated (Fig. 8F and G). Meanwhile, SENP6-deficient mice showed an increased lung wet-to-dry (W/D) ratio (Fig. 8H), and hematoxylin and eosin (H&E) staining of lung tissue revealed aggravated inflammatory cell infiltration (Fig. 8I and J). We further observed that NLRP3 protein levels were elevated in the lungs of SENP6*^fl/fl^ Lyz2-cre* mice following LPS stimulation, whereas the mRNA levels of *Nlrp3* and *Il1b* remained unchanged (Fig. 8K to N). Consistently, immunofluorescence staining revealed increased expression of NLRP3 and IL-1β in lung tissues of SENP6*^fl/fl^ Lyz2-cre* mice (Fig. 8O and P).

We also assessed SENP6’s role in NLRP3-driven peritonitis via intraperitoneal alum injection. Compared to SENP6*^fl/fl^* mice, SENP6-deficient mice exhibited elevated serum levels of IL-1β and IL-18 (Fig. S5C and D), with no significant change in TNF-α levels (Fig. S5E). In addition, alum stimulation markedly increased IL-1β and IL-18 production in the peritoneal lavage fluid of SENP6-deficient mice (Fig. S5F and G), while TNF-α levels remained unchanged (Fig. S5H). H&E staining revealed exacerbated inflammatory features in the lungs of SENP6-deficient mice (Fig. S5I and J). Additionally, we employed an intratracheal LPS instillation-induced ALI model. The results showed that NLRP3 expression was significantly increased in the lung tissue of SENP6 knockout mice (Fig. S5K). Furthermore, the lung W/D ratio was also significantly higher in SENP6 knockout mice compared to SENP6*^fl/fl^* mice (Fig. S5L). In parallel, serum and BALF levels of IL-1β and IL-18 were significantly elevated in SENP6*^fl/fl^* Lyz2-cre mice, with no significant change in TNF-α levels (Fig. S5M to R) Taken together, these findings indicate that SENP6 deficiency aggravates NLRP3-dependent inflammation in vivo.

## Discussion

Dysregulated NLRP3 inflammasome activity contributes to the pathogenesis of diverse inflammatory conditions, including autoimmune disorders, cancer, respiratory diseases, cardiovascular dysfunction, neurodegeneration, and metabolic syndromes [1,13,31,32]. ALI, characterized by respiratory distress and refractory hypoxemia, exhibits high morbidity and mortality. NLRP3 inflammasome activation significantly contributes to ALI progression by intensifying inflammatory responses and worsening disease severity [33]. Therefore, precise control of NLRP3 inflammasome activity is critical to mitigate excessive inflammation and prevent tissue damage.

Here, we demonstrate that SENP6 negatively regulates the priming and activation of the NLRP3 inflammasome by specifically removing SUMO2/3 chains from NLRP3, and reveal the potential mechanism by which SENP6 inhibits NLRP3 inflammasome activation: (a) SENP6 directly binds to the PYD and NACHT domains of NLRP3. (b) SENP6 deSUMOylates NLRP3 at K23, K204, and K689, recruiting MARCHF7 to selectively promote K48-linked ubiquitination of NLRP3, thereby facilitating its autophagic degradation and inhibiting NLRP3 stability and protein expression. (c) Phenotypically, SENP6 knockout significantly exacerbates LPS-induced ALI and alum-induced peritonitis.

Our findings reveal a temporally coordinated regulatory network in which SENP6 fine-tunes NLRP3 inflammasome activation through dual-phase modulation. In the priming phase, SENP6 deficiency enhances ERK and JNK phosphorylation following LPS stimulation, suggesting that SENP6 normally restrains MAPK-driven transcription of inflammasome components such as NLRP3 and pro-IL-1β. During the subsequent activation phase, SENP6 expression increases and directly interacts with NLRP3 to remove SUMO2/3 chains at K23, K204, and K689, thereby recruiting MARCHF7 to catalyze K48-linked polyubiquitination and promote autophagic degradation of NLRP3. These sequential events indicate that SENP6 couples early inflammatory priming with late-phase resolution, forming a self-limiting feedback loop. Early MAPK activation acts as an “accelerator” for rapid priming, while the later SENP6–MARCHF7-mediated degradation functions as a “brake”, preventing prolonged inflammasome activation. This temporal interplay highlights SENP6 as a key integrator linking MAPK signaling with ubiquitin–autophagy-mediated control of NLRP3 homeostasis.

Mounting evidence suggests that different types of PTMs dynamically determine NLRP3 stability and activity through site competition on the same substrate. In the resting state, NLRP3 is at least partially maintained in an inactive conformation by PTMs. Upon stimulation, rapid dynamic changes in various PTMs up-regulate NLRP3 protein levels to tightly control optimal NLRP3 activation [11], but the interaction mechanisms among NLRP3’s various PTMs remain unclear. Among these modifications, SUMOylation critically influences NLRP3 stability and activity. Previous studies have shown that the E3 SUMO ligase TRIM28 promotes NLRP3 inflammasome activation by SUMOylating NLRP3, which prevents its K48-linked ubiquitination and degradation, thereby enhancing NLRP3 stability and promoting inflammasome activation [12]. However, Barry et al. [7] reported that SENP6/SENP7 desumoylation of NLRP3 promotes inflammasome activation, suggesting that desumoylation by SENP6 acts as a positive regulator.

The discrepancies between our findings and those of Barry et al. may stem from key differences in experimental design. Barry et al. primarily used bone marrow-derived macrophages (BMDMs), while we employed MH-S cells and primary PMs, which may have distinct immune responses and regulatory mechanisms for SENP6. Additionally, while Barry et al. focused on in vitro models, our study utilized in vivo models of ALI and peritonitis, offering a more complex, physiologically relevant context for observing SENP6’s role in NLRP3 regulation.

Furthermore, Barry et al. concentrated on the early desumoylation events in NLRP3 activation, whereas our study focuses on the long-term effects of SENP6 on NLRP3 stability and degradation. The extended time frame in our study allowed us to capture the sustained influence of SENP6 in controlling NLRP3 degradation, in contrast to the transient desumoylation effects observed by Barry et al.

Our data extend this framework by demonstrating that SENP6 expression is up-regulated during NLRP3 inflammasome activation. In SENP6-deficient cells, SUMO2/3 conjugation of NLRP3 is markedly increased, indicating that elevated SENP6 normally counteracts SUMO2/3 accumulation on NLRP3. This increased SENP6 promotes deSUMOylation of NLRP3 at residues K23, K204, and K689, thereby facilitating MARCHF7-mediated K48-linked polyubiquitination and subsequent autophagy lysosome-dependent degradation. Thus, SENP6 restrains excessive NLRP3 protein accumulation and inflammasome activity through a deSUMOylation to ubiquitination switch, revealing a critical crosstalk mechanism between SUMOylation and ubiquitination that limits inflammasome activation.

We further show that the PYD and NACHT domains are critical for SENP6-mediated deSUMOylation. The PYD domain primarily mediates PYD–PYD interactions between NLRP3 and ASC, initiating the assembly process [34], SENP6 binding here may hinder the initial steps of inflammasome assembly. Meanwhile, the NACHT domain possesses ATPase activity that drives NLRP3 self-oligomerization and conformational changes [34]. SENP6 interaction may inhibit this enzymatic activity or oligomerization potential, maintaining NLRP3 in an inactive state. This dual interference may not only directly weaken NLRP3 activation but also promote recognition of degradation signals through induced conformational changes, thereby enhancing subsequent autophagy–lysosome pathway mediation. Mutagenesis studies confirm that K23 (PYD), K204 (NACHT), and K689 (linker between NACHT and LRR) are essential for deSUMOylation of NLRP3. The precise role of each lysine in this process, however, is complex. K23 is located within the PYD domain, a key site for interaction with ASC, and SUMOylation at this position may modulate the ability of NLRP3 to undergo oligomerization by hindering its interaction with ASC. K204, within the NACHT domain, is involved in self-oligomerization and ATP-driven conformational changes. SUMOylation at this site may act as a switch to either promote or inhibit NLRP3’s active form. K689, located between NACHT and LRR, is a critical regulatory hotspot, with dynamic SUMOylation and ubiquitination at this site dictating NLRP3’s fate. The divergent outcomes of SUMOylation mediated by distinct enzymes are likely related to site-specific lysine targeting. Systematic mapping of the dynamic SUMOylation and ubiquitination profiles at individual residues during NLRP3 inflammasome signaling will be essential to fully elucidate the underlying regulatory mechanisms.

MARCHF7, an E3 ligase previously shown to mediate dopamine-induced inhibition of the NLRP3 inflammasome via K48-linked ubiquitination and autophagic degradation [35], also polyubiquitinates ATG14 to regulate autophagy flux [36]. Given SENP6’s exclusive deSUMOylase activity, we hypothesized that it modulates NLRP3 ubiquitination by recruiting an E3 ligase. Our observation that SENP6 promotes K48-linked ubiquitination and autophagic degradation of NLRP3 paralleled prior reports of the deubiquitinase USP5 scaffolding MARCHF7 for similar outcomes [30], prompting us to prioritize MARCHF7.

As a member of the MARCH E3 ligase family, MARCHF7 exhibits unique SENP6-dependent regulatory features, distinct from those of MARCH2 and MARCH5, which have also been reported to promote NLRP3 degradation via K48-linked ubiquitination [37,38]. However, MARCHF7 exhibits distinct regulatory features in this pathway. Unlike MARCH2 or MARCH5, its recruitment to NLRP3 strictly depends on SENP6-mediated deSUMOylation at K23, K204, and K689. SENP6’s enzymatic activity enhances the interaction between MARCHF7 and NLRP3, forming a regulatory cascade that integrates SUMOylation–ubiquitination crosstalk. This mechanism has not been reported for other MARCH family members. This specificity underscores MARCHF7’s unique role as a SENP6-dependent node in the posttranslational network governing NLRP3 stability.

Additionally, the potential interplay between MARCHF7 and TRIM31, another E3 ligase mediating K48-linked NLRP3 polyubiquitination, warrants consideration [26]. These ligases may exert redundant or complementary effects, with TRIM31 operating independently of SUMOylation, while MARCHF7 requires SENP6-triggered deSUMOylation for efficient engagement. Their cooperation could be spatially, temporally, or stimulus-dependent, highlighting multilayered control of NLRP3 degradation. Future studies exploring MARCHF7–TRIM31 interactions will clarify whether they function redundantly or in parallel pathways.

Nevertheless, this study has several limitations, which point the way for future research. Firstly, although we revealed that SENP6 initiates the K48-linked ubiquitination degradation program of NLRP3 at K23, K204, and K689 sites by deSUMOylation, the dynamic temporal competition between SUMOylation and ubiquitination at these sites remains unresolved. Secondly, how SENP6 specifically affects the autophagy process and whether there are other intermediate proteins involved in this SUMO ubiquitin crosstalk, the precise molecular mechanism of which still needs further clarification. Finally, our experiment mainly focused on macrophages, and further validation is needed to determine whether the SENP6–MARCHF7 axis follows the same regulatory logic in other key cell types expressing NLRP3, such as neutrophils or epithelial cells.

Translationally, our findings highlight the SENP6–MARCHF7 axis as a degradative pathway that could be leveraged for the design of targeted chimeric degraders such as proteolysis-targeting chimera (PROTACs). By recruiting NLRP3 to MARCHF7 for ubiquitination or mimicking the SENP6–NLRP3 interaction, such strategies could achieve precise and efficient degradation of NLRP3. This approach offers a promising therapeutic paradigm for inflammatory diseases that currently lack specific NLRP3 inhibitors. Compared with conventional small-molecule inhibitors, PROTAC-based degraders may provide sustained efficacy, reduced drug resistance, and the ability to target otherwise “undruggable” proteins, representing an exciting avenue for treating NLRP3-driven pathologies [39].

In conclusion, SENP6 suppresses NLRP3 inflammasome activation by removing SUMO2/3 modifications at K23, K204, and K689, recruiting MARCHF7 to promote K48-linked ubiquitination, and driving autophagy–lysosome-mediated degradation. By lowering NLRP3 protein levels and preventing inflammasome assembly, SENP6 functions as a brake on excessive inflammatory responses. SENP6 deficiency in vivo exacerbates LPS-induced endotoxic shock-associated lung injury and alum-induced peritonitis. Therefore, SENP6 may serve as a potential therapeutic target for NLRP3 inflammasome-related diseases.

## Materials and Methods

### Animals

C57BL/6J mice (male, aged 6 to 8 weeks) were sourced from Charles River (MA, USA). SENP6-floxed mice (C57BL/6J background, SENP6*^fl/fl^*) and Lyz2-cre mice were purchased from Cyagen (Suzhou, China). SENP6*^M-KO^* mice (SENP6*^fl/fl^ Lyz2-cre*) were hybridized from SENP6*^fl/fl^* and Lyz2-cre mice. All animals were housed under specific pathogen-free (SPF) conditions, maintained at 50 ± 5% humidity and 22 ± 2 °C, with a 12-h light/dark cycle. All procedures involving animals were approved by the Ethics Review Committee of Shandong First Medical University.

### Cell culture

The murine alveolar macrophage cell line (MH-S) and the human embryonic kidney cell line (HEK293T) were obtained from Procell (Wuhan, China). MH-S cells were cultured in RPMI 1640 medium (Procell), while HEK293T cells were maintained in high-glucose (4.5 g/l) Dulbecco’s modified Eagle’s medium (DMEM; Procell). All culture media were supplemented with 10% fetal bovine serum (FBS; Lonsera, Suzhou, China) and 1% penicillin–streptomycin (P/S; Cytiva, NY, USA). Cells were incubated at 37 °C in a humidified atmosphere containing 5% CO₂.

Mouse PMs were obtained by intraperitoneal injection of 3% thioglycolate (MCE, HY-W115724, NJ, USA). After 4 d, peritoneal exudate macrophages were collected and seeded into culture plates. The following day, the medium was replaced with fresh DMEM supplemented with 10% FBS and 1% P/S. The adherent monolayer cells were used as mouse PMs [40].

THP-1 cells (FuHeng Biology, Shanghai, China) were differentiated into macrophages by treatment with 100 nM phorbol 12-myristate 13-acetate (PMA) (Sigma-Aldrich, 19-144, St. Louis, USA) for 24 h.

### Reagents

LPS (*Escherichia coli*, O55: B5, L2880), ATP (A3377), and anti-SENP6 (QC22727) were purchased from Sigma-Aldrich (St. Louis, USA). MG132 (S2619), CHX (S7418), CQ (S6999), 3-MA (S2767), and carfilzomib (PR-171) were purchased from Selleck (TX, USA). Nigericin (28643-80-3), alum (21645-51-2), and poly(dA:dT) (86828-69-5) were from InvivoGen (San Diego, USA). Disuccinimidyl suberate crosslinker (HY-W019543), U0126 (HY-12031A), and SP600125 (HY-12041) were purchased from MCE (Shanghai, China). Anti-NLRP3 (15101S), anti-caspase-1/p20 (3866S), anti-IL-1β/p17 (63124S), anti-NLRC4 (12421S), and anti-AIM2 (12948S) were from Cell Signaling Technology (MA, USA). Anti-β-actin (66009-1-Ig), anti-hemagglutinin (HA) (51064-2-Ap), anti-His (66005-1-Ig), anti-DDDDK/Flag (66008-3-Ig), anti-SUMO1 (10329-1-Ap), anti-SUMO2/3 (11251-1-Ap), anti-p65 (10745-1-Ap), anti-pp65 (82335-1-RR), anti-IκBα (10268-1-AP), anti-p-IκBα (82349-1-RR), anti-ERK1/2 (11257-1-AP), anti-p-ERK1/2 (28733-1-AP), anti-JNK (24164-1-AP), and anti-p-JNK (80024-1-RR) were purchased from Proteintech Group (Wuhan, China). Anti-ASC (R013826) and anti-Myc (LF-301S) were purchased from Epizyme Biotech (Shanghai, China). Anti-MARCHF7 was purchased from Santa Cruz Biotechnology (CA, USA).

### Plasmid construction and transfection

Myc-SENP6, Myc-SENP6-ΔN domain, Myc-SENP6-ΔPeptidase1, Myc-SENP6-ΔPeptidase2, Myc-SENP6^C1030S^, His-MARCHF7, and point mutant (K23R, K26R, K204R, K689R, K942R, and K1025R) plasmids were purchased from Abiotech (Jinan, China). Flag-NLRP3, Flag-NLRP3-ΔPYD, Flag-NLRP3-ΔNACHT, Flag-NLRP3-ΔLRR, Flag-CASP1, Flag-PYCARD, HA-SUMO2, and HA-SUMO3 plasmids and the plasmids for HA-tagged WT ubiquitin or its mutants (K27, K48, K48R, and K63) were purchased from BioSune Biotechnology (Shanghai, China). All constructs were confirmed by DNA sequencing. Plasmids were transiently transfected into HEK293T cells with Lipofectamine 3000 reagent (Invitrogen, USA) according to the manufacturer’s instructions.

### RNA extraction and quantitative real-time PCR

Total RNA was extracted using the Fastagen RNA Extraction Kit (Shanghai, China), and cDNA was synthesized with the HiScript III RT SuperMix for quantitative polymerase chain reaction (qPCR) (+gDNA wiper) kit from Vazyme (R323, Nanjing, China), following the manufacturer’s protocol. Quantitative real-time PCR (RT-qPCR) was performed using the SYBR Green assay (Vazyme, Q713) on the CFX96 Touch Real-Time PCR Detection System (Bio-Rad, CA, USA). Expression levels were normalized to the mRNA level of *β-actin*. Each sample was analyzed in triplicate, and relative expression levels were calculated using the 2−ΔΔ*C*_t_ method. The specific primers used for RT-qPCR are listed in Table S2.

### Western blot analysis

Proteins from cells and tissues were extracted by lysing with radioimmunoprecipitation assay (RIPA) buffer (Beyotime, P0013D) supplemented with a protease inhibitor cocktail (Servicebio, G2006-250UL). After 15 min of centrifugation at 12,000 rpm at 4 °C, protein concentrations in the lysates were determined using a bicinchoninic acid (BCA) protein assay kit (Sevenbio, SW101). Protein samples were separated by 10% sodium dodecyl sulfate–polyacrylamide gel electrophoresis (SDS-PAGE) and transferred onto polyvinylidene difluoride (PVDF) membranes (Millipore, C3117). Following a 1-h blocking step with 5% bovine serum albumin (BSA) (Seven Biotech, SO110), the membranes were incubated with primary antibodies overnight at 4 °C. Subsequently, the membranes were incubated with secondary antibodies (ZSGB-bio, ZB-2301 and ZB-2305) for 1 h. Protein signals were detected using the Amersham ImageQuant 800 Western blot imaging system (Cytiva, USA), and chemiluminescence was developed using the Omni-ECL Western Blotting Substrate (Epizyme Biotech, SQ203). Quantitative analysis of the blots was performed using ImageJ software [National Institutes of Health (NIH), USA].

### CO-IP assay

Cells were lysed using Pierce lysis buffer (25 mM tris–HCl, pH 7.4, 150 mM NaCl, 1 mM EDTA, 1% NP-40, and 5% glycerol; Thermo Fisher Scientific, 87788) supplemented with a protease inhibitor cocktail (Servicebio, G2006-250UL). Protein A/G magnetic beads (Selleck, B23202) were pre-washed 4 times with the lysis buffer. The cell lysate was then incubated with the appropriate primary antibodies overnight at 4 °C, followed by the addition of protein A/G magnetic beads for 4 h. After washing the beads 5 times with the lysis buffer, the immunoprecipitated proteins were eluted with 1× SDS-PAGE loading buffer and subsequently analyzed by Western blotting.

### Immunofluorescent confocal analysis

Cells plated on cover glasses were washed with ice-cold phosphate-buffered saline (PBS), fixed with 4% paraformaldehyde (Beyotime, P0099), and permeabilized with 0.5% Triton X-100 (Beyotime, P0096) for 10 min at room temperature. Following permeabilization, cells were blocked with 5% BSA for 1 h at room temperature. The cells were then incubated overnight at 4 °C with primary antibodies: anti-NLRP3 (1:600, Cell Signaling Technology) and anti-IL-1β (1:600, Cell Signaling Technology). After primary antibody incubation, cells were incubated with species-matched fluorophore-conjugated secondary antibodies for 1 h at room temperature. Following 3 washes, the nuclei were stained with 4′,6-diamidino-2-phenylindole (DAPI) (Abcam, ab104139). Cells were subsequently visualized and analyzed using a TI-2 fluorescence microscope (Nikon, Tokyo, Japan).

To assess the colocalization of NLRP3 with SENP6, NLRC4, or AIM2, PMs cells were plated on confocal dishes. Cells were incubated with the appropriate primary antibodies and then stained with fluorophore-conjugated secondary antibodies for 1 h at room temperature. Colocalization was visualized using a TI-2 fluorescence microscope (Nikon, Tokyo, Japan) and analyzed with ImageJ software.

For ASC speck staining, PMs were plated on cover glasses in 6-well plates. After stimulation with LPS (200 ng/ml, 4 h) and ATP (2 mM, 45 min) or nigericin (10 μM, 1 h) or poly(dA:dT) (2 μg/ml, 6 h), the cells were processed as described above. ASC specks were then visualized using a TI-2 fluorescence microscope (Nikon, Tokyo, Japan).

### ASC oligomerization assay

To detect the oligomerization of ASC, PMs were stimulated with LPS (200 ng/ml, 4 h), followed by ATP (2 mM, 45 min) or nigericin (10 μM, 1 h). After stimulation, the medium was discarded, and the cells were washed with cold PBS. The cells were then incubated with 500 μl of RIPA buffer at 4 °C for 10 min. Following incubation, the cells were scraped off and centrifuged at 6,000*g* for 15 min at 4 °C, and the supernatant (50 μl) was collected for Western blot analysis. The pellets were then washed with ice-cold PBS, resuspended in 500 μl of PBS, and treated with 2 mM disuccinimidyl suberate crosslinker [dissolved in dimethyl sulfoxide (DMSO)] for 30 min at room temperature. After crosslinking, the samples were centrifuged at 6,000*g* for 15 min. The crosslinked pellet was resuspended in 40 μl of SDS loading buffer, boiled at 100 °C for 10 min, and then analyzed by Western blotting.

### ELISA and LDH assay

Cell culture supernatants, animal serum, BALF, and peritoneal lavage fluid (PLF) were collected to measure the protein levels of IL-1β (Reed Biotech, RE1074M), IL-18 (Reed Biotech, RE1123M), and TNF-α (Reed Biotech, RE1060M) using commercially available enzyme-linked immunosorbent assay (ELISA) kits. Cytotoxicity was assessed using an LDH assay kit (Beyotime, C0018S) according to the manufacturer’s protocol.

### RNA interference

MH-S or HEK293T cells were plated in 6-well plates and cultured for 12 h before transfection. Cells were then transfected with 100 nM siRNA using Lipofectamine 3000 (Invitrogen, USA) following the manufacturer’s instructions. The siRNAs were synthesized by Gene Pharma (Shanghai, China), and the sequences of the siRNAs used are provided in Table S3.

### In vivo mouse model of LPS-induced endotoxemia

To assess LPS-induced endotoxemia, 6-week-old SENP6*^fl/fl^* and SENP6*^fl/fl^ Lyz2-cre* mice received intraperitoneal injections of LPS (25 mg/kg) or saline. After 12 h, blood samples were collected from the retro-orbital vein. Plasma was separated by centrifugation at 500*g* for 15 min at 4 °C and used for ELISA. Following blood collection, mice were euthanized under anesthesia via exsanguination. BALF and lung tissues were then harvested for further analyses. NLRP3 protein levels in freshly isolated lung tissues were measured by immunoblotting.

### In vivo mouse model of intratracheal LPS-induced ALI

A mouse LPS-induced ALI model was established as previously described [41]. Briefly, after anesthesia, mice were subjected to intratracheal instillation of LPS (2 mg/50 μl). The control group received physiological saline. Mice were euthanized under anesthesia via exsanguination 24 h later, and plasma, BALF, and lung tissue were collected from each group.

### H&E staining and lung injury scoring

Morphological changes in lung tissue were evaluated by fixing specimens in 4% paraformaldehyde (Servicebio, G1101), embedding them in paraffin, and cutting sections 5 μm thick. H&E staining was performed by Servicebio (Wuhan, China). ALI scoring was assessed from 3 randomly selected, nonoverlapping fields per section using a digital slide scanner (VS200, Olympus), and the mean score was calculated.

Lung injury severity was quantified using an ALI scoring system comprising 4 parameters: (a) inflammatory cell infiltration (0 to 5), (b) alveolar edema (0 to 5), (c) alveolar hemorrhage (0 to 5), and (d) septal hemorrhage and congestion (0 to 5) [42]. The total ALI score was the sum of the 4 parameter scores. All histological evaluations were conducted in a blinded manner to ensure objectivity.

### BALF collection and analysis

BALF was collected by performing tracheostomy and tracheal intubation, followed by 3 sequential instillations of pre-cold sterile PBS (total volume, 3 ml) into the bronchoalveolar space. The fluid was gently withdrawn, and the recovered BALF was centrifuged at 400*g* for 10 min at 4 °C. The supernatant was stored at −80 °C for subsequent analysis, while the cell pellet was counted. Protein concentrations in the supernatant were determined using a BCA assay kit.

### Lung wet/dry ratio

Pulmonary edema severity was evaluated using the W/D weight ratio. The right lung was excised, weighed to obtain the wet weight (W), and dried in a constant-temperature oven at 80 °C for 48 h until a stable weight was reached, recorded as the dry weight (D). The W/D ratio was then calculated.

### In vivo mouse model of alum-induced peritonitis

The alum-induced peritonitis model was generated by intraperitoneal injection of 700 μg of alum. After 12 h, retro-orbital blood was collected for serum ELISA. Mice were euthanized under anesthesia via exsanguination, and lungs were harvested for H&E staining to evaluate histological changes. The peritoneal cavity was then lavaged with 4 ml of cold sterile PBS, and the lavage fluid was centrifuged at 500*g* for 5 min. The resulting supernatant was concentrated for ELISA analysis.

### Statistical analysis

All statistical analyses were performed using GraphPad Prism software version 9.0 (GraphPad Software, USA). Data are expressed as the mean ± standard deviation (SD) from at least 3 independent experiments, rather than technical duplications. Statistical analyses were conducted using appropriate methods, including the 2-tailed Student’s *t* test, one-way analysis of variance (ANOVA), and 2-way ANOVA, depending on the specific requirements of the data. A *P* value less than 0.05 was considered statistically significant. Statistical significance is denoted as follows: *P < 0.05, **P < 0.01, ***P < 0.001, ****P < 0.0001, and ns means no significance.

## Ethical Approval

All mice were housed in an SPF facility at the Center for Medical and Technological Innovation of Shandong First Medical University. The animal experiments were approved by the Institutional Animal Policy and Welfare Committee of Shandong First Medical University and was conducted in strict accordance with institutional guidelines.

## Data Availability

All data are available in the main text or the Supplementary Materials.
