# Supplementary material for: SENP6 Restrains NLRP3 Inflammasome Activation via DeSUMOylation-Driven K48-Linked Ubiquitination of NLRP3 in Acute Lung Injury
Source: Research (Wash D C). 2026 Jan 12;9:1069. doi: 10.34133/research.1069 (PMC12794193; doi:10.34133/research.1069)
Supplement: Supplementary 1 — Figs. S1 to S6 Tables S1 to S3 [file research.1069.f1.docx]

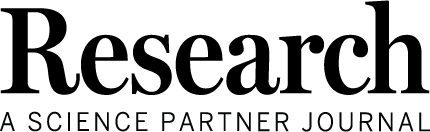


Supplementary Materials for

**SENP6 Restrains NLRP3 Inflammasome Activation via DeSUMOylation-Driven K48-Linked Ubiquitination of NLRP3 in Acute Lung Injury**

Angran Gu and Bailun Wang *et al.*

*Corresponding author:Yuelan Wang Email: LXQ9066@163.com

**This file includes:**

Figures. S1 to S6 and Figure legends

Tables S1 to S3

**
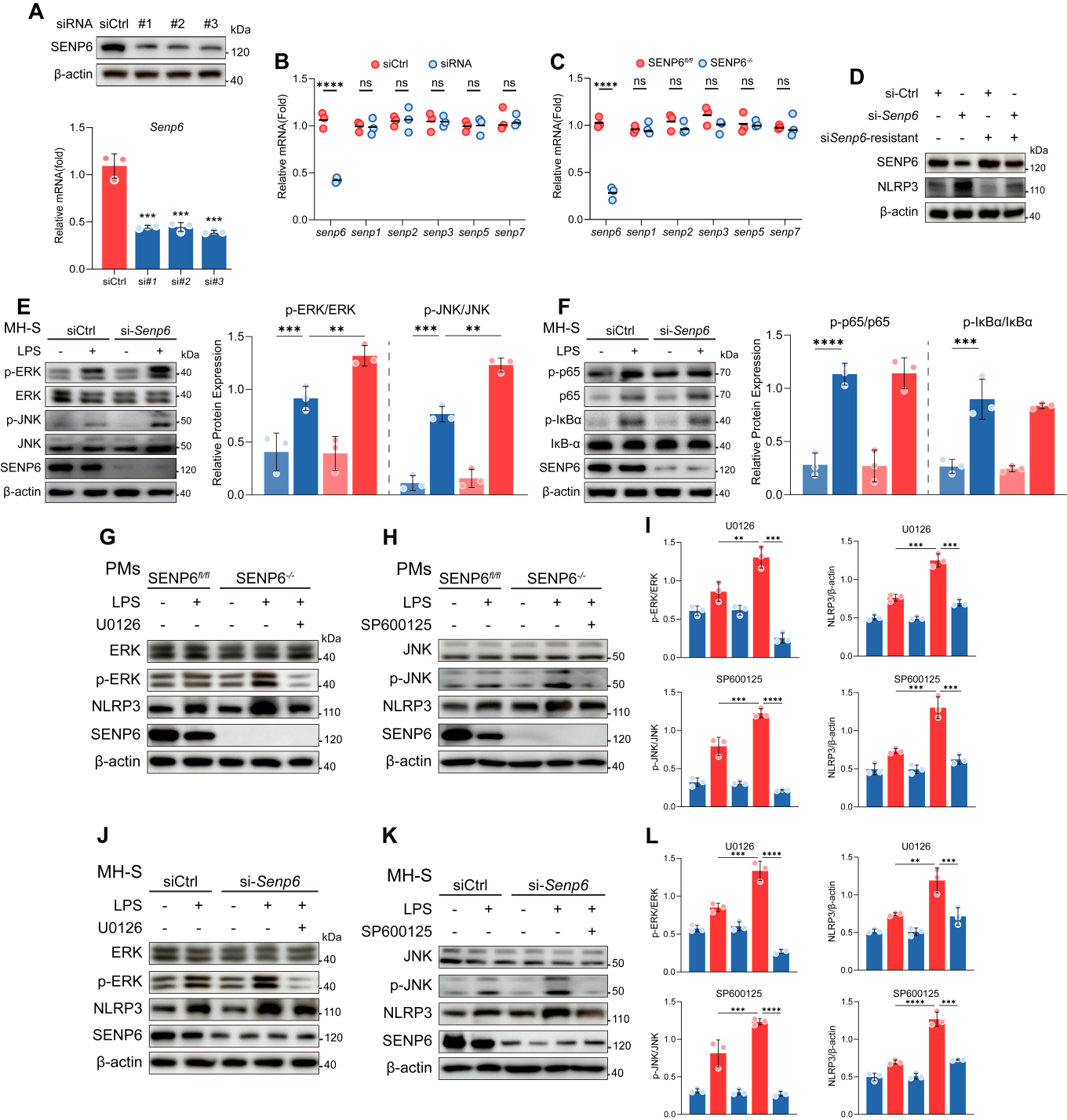
**

**Figure S1. SENP6 deficiency enhances MAPK pathway activation following LPS treatment. (A, B)** Efficiency of SENP6 knockdown in murine alveolar macrophages (MH-S) transfected with control siRNA (si-Ctrl) or SENP6-targeting siRNA (si-*Senp6*) for 48 h. Knockdown efficiency was confirmed by quantifying SENP6 mRNA levels via qPCR and SENP6 protein expression by immunoblotting (n=3). **(B, C)** Quantitative analysis of mRNA expression levels of SENP family members in MH-S cells transfected with si-*Senp6* and SENP6*^fl/fl^ Lyz2-cre* mice lung tissue (n=3). **(D)** MH-S cells were transfected with si-Ctrl or si-*Senp6* together with the si*Senp6*-resistant construct, and whole-cell lysates were subsequently analyzed by immunoblotting. **(E-F)** Immunoblot analysis of phosphorylated and total ERK, JNK, p65, and IκBα in WCL from LPS-stimulated MH-S cells transfected with si-Ctrl or si-*Senp6* (n=3). **(G-I)** SENP6*^fl/fl^* and SENP6*^fl/fl^* *Lyz2-cre* PMs were stimulated with LPS for 4 h, followed by treatment with the ERK inhibitor U0126 (10 μM) or the JNK inhibitor SP600125 (10 μM) for an additional 1.5 h. **(G)** Representative immunoblots showing total and phosphorylated ERK or JNK, NLRP3, SENP6, and β-actin **(H)**, and relative protein levels were quantified **(I)**. **(J-L)** MH-S cells were transfected with control si-Ctrl or si-*Senp6* and then stimulated with LPS for 4 h, followed by treatment with either the ERK inhibitor U0126 (10 μM) **(J)** or the JNK inhibitor SP600125 (10 μM) **(K)** for an additional 1.5 h. Protein levels of total and phosphorylated ERK or JNK, NLRP3, SENP6, and β-actin were determined by immunoblotting, and relative protein levels were quantified **(L)** (n=3). Data are presented as mean ± SD. Statistical analysis was performed using Student’s t-test (A) or Two-way ANOVA with Bonferroni test, based on n = 3 independent biological experiments. ***P* < 0.01, ****P* < 0.001, *****P* < 0.0001 and ns means no significance.

**
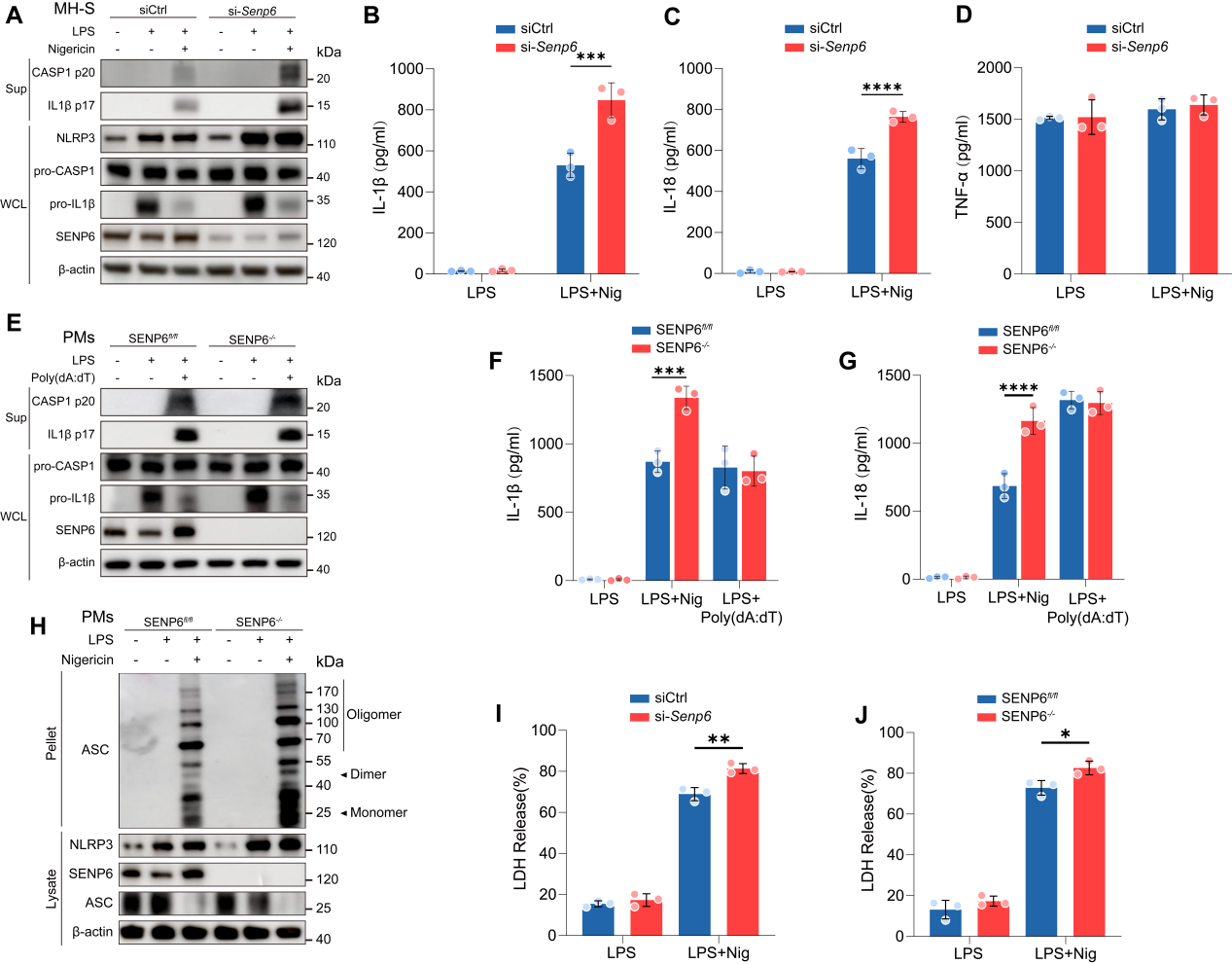
**

**Figure S2. SENP6 deficiency promotes NLRP3 inflammasome activation. (A)** Immunoblot analysis of SN and WCL from MH-S cells transfected with si-Ctrl or si-*Senp6*, followed by LPS priming and Nigericin activation. **(B-D)** ELISA quantification of IL-1β **(B)**, IL-18 **(C)**, and TNF-α **(D)** levels in SN from LPS-primed, Nigericin-activated MH-S cells transfected with si-Ctrl or si-*Senp6* (n=3). **(E)** Immunoblot analysis of SN and WCL from LPS-primed, poly(dA:dT)-activated (2 μg/ml, 6 h) PMs isolated from SENP6*^fl/fl^* and SENP6*^fl/fl^ Lyz2-cre* mice. **(F, G)** ELISA measurement of IL-1β **(F)** and IL-18 **(G)** secretion in SN from LPS-primed SENP6*^fl/fl^* and SENP6*^fl/fl^ Lyz2-cre* PMs activated with Nigericin or poly(dA:dT) (n=3). **(H)** Immunoblot analysis of ASC oligomerization in pellet fractions and WCL from LPS-primed, nigericin-activated SENP6*^fl/fl^* and SENP6*^fl/fl^ Lyz2-cre* PMs. **(I, J)** LDH release in SN from MH-S cells transfected with si-Ctrl or si-*Senp6*, and from PMs of SENP6*^fl/fl^* and SENP6*^fl/fl^ Lyz2-cre* mice, following LPS priming and Nigericin activation (n=3). Data are presented as mean ± SD. Statistical analysis was performed using Two-way ANOVA with Bonferroni test, based on n = 3 independent biological experiments. **P* < 0.05, ***P* < 0.01, ****P* < 0.001, *****P* < 0.0001.

**
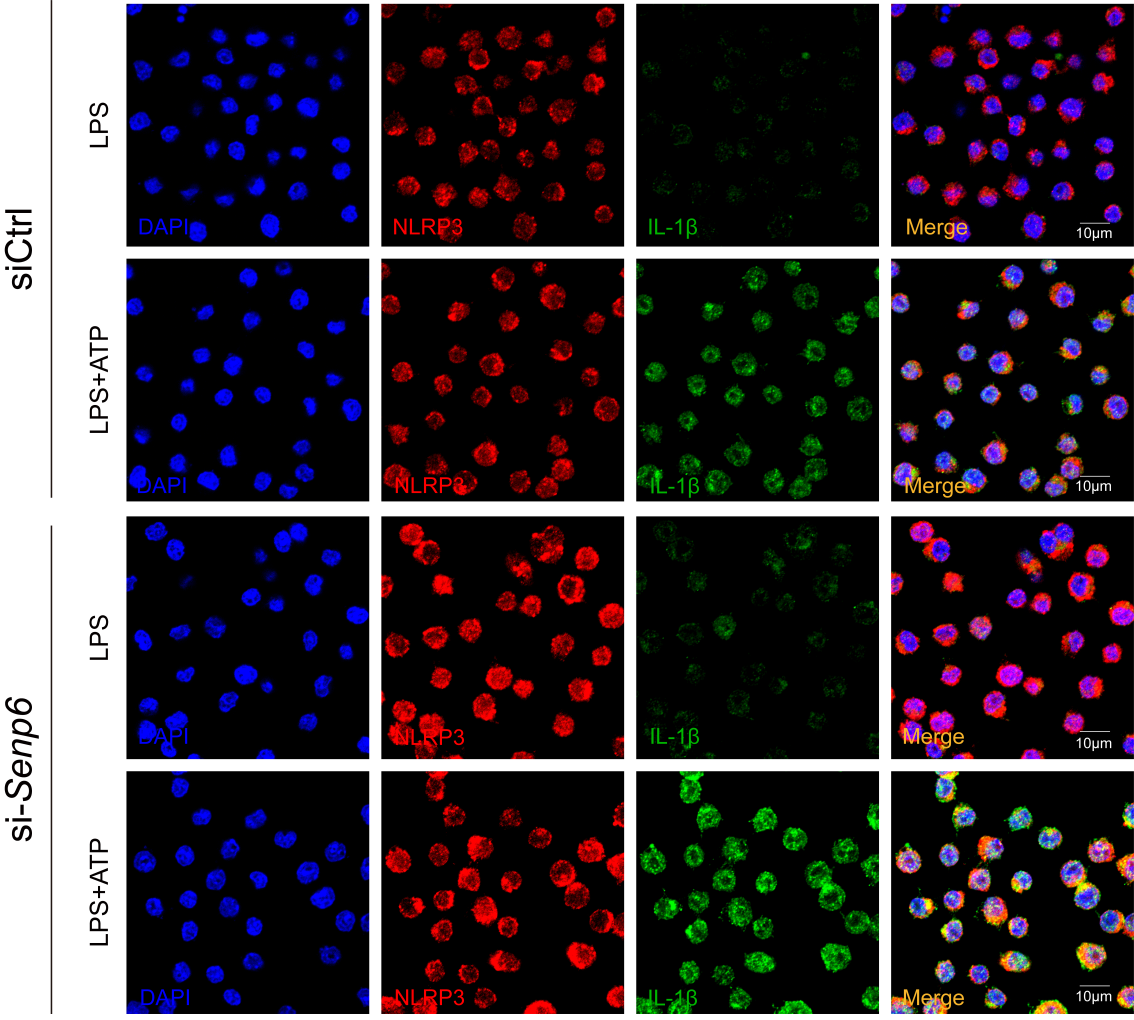
**

**Figure S3. SENP6 knockdown reduces NLRP3 and IL-1β expression.**

Representative confocal images of MH-S cells transfected with si-Ctrl or si-*Senp6*, following LPS priming and subsequent ATP activation. NLRP3, red; IL-1β, green; nuclei, blue. Scale bar, 10 μm.

**
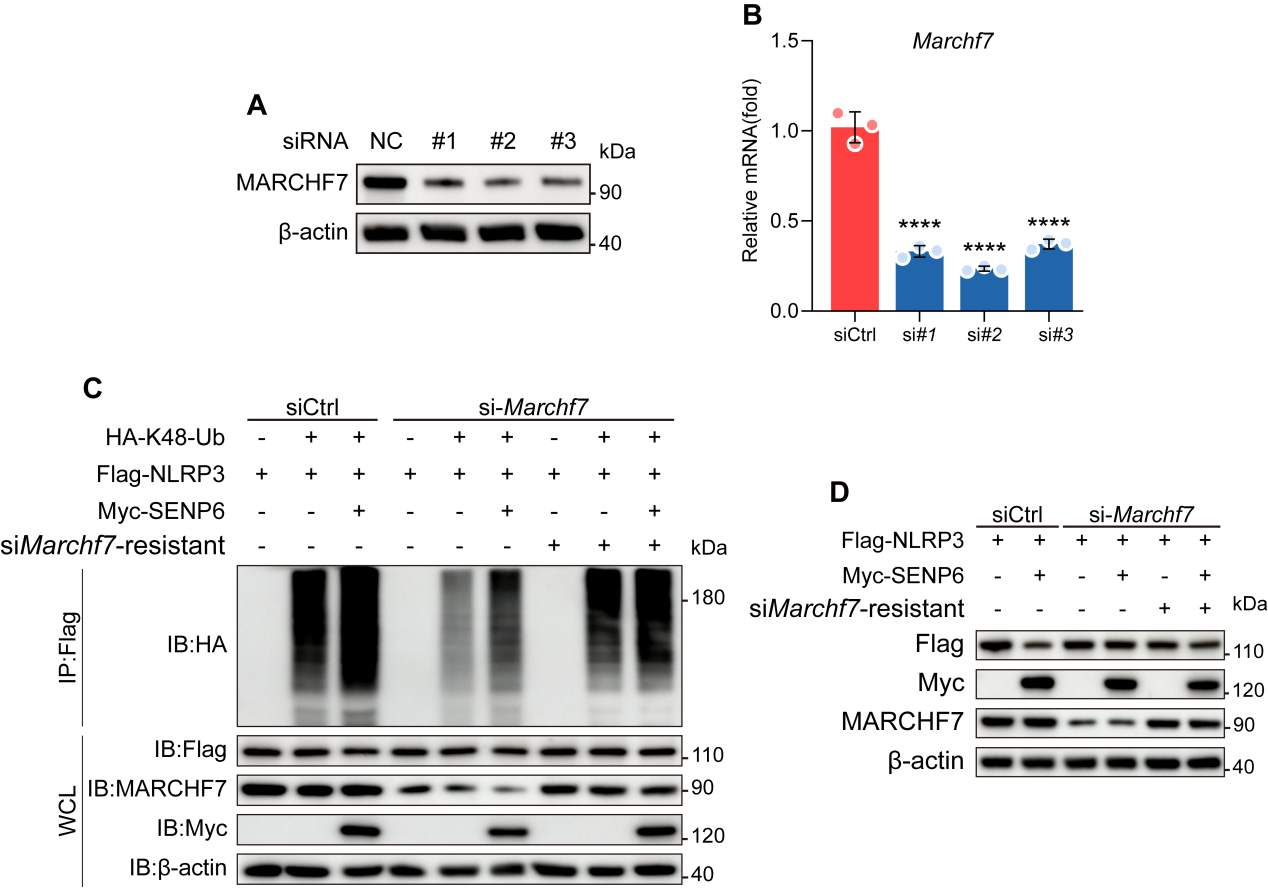
**

**Figure S4. Validation of MARCHF7 Knockdown Efficiency and Functional Specificity.**

**(A, B)** Immunoblot and qPCR analysis of cell lysates from MH-S cells transfected with si-Ctrl or si-*Marchf7* #1, #2, or #3 (n=3). **(C)** HA-K48-Ub, Flag-NLRP3, Myc-SENP6 and si*Marchf7*-resistant were co-transfected into control or *Marchf7*-knockdown HEK293T cells, lysates were immunoblotted and immunoprecipitated with anti-HA. **(D)** Flag-NLRP3, Myc-SENP6 and si*Marchf7*-resistant were co-transfected into control or *Marchf7*-knockdown HEK293T cells. Whole-cell lysates were subsequently analyzed by immunoblotting. Data are presented as mean ± SD. Statistical analysis was performed using One-way ANOVA with Bonferroni test, based on n = 3 independent biological experiments. ***P* < 0.01, ****P* < 0.001, *****P* < 0.0001 and ns means no significance.

**
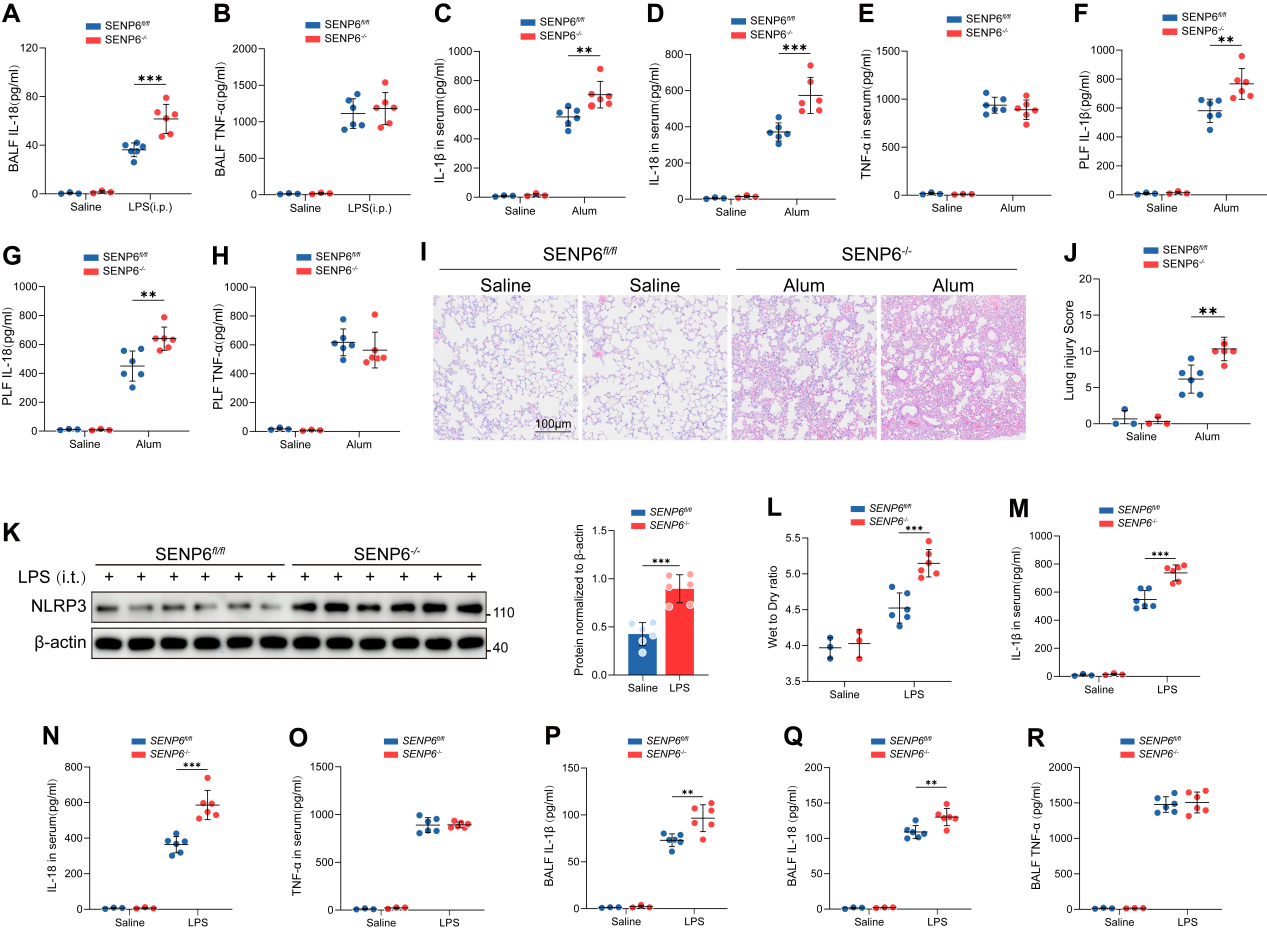
**

**Figure S5. SENP6 deficiency promotes inflammation in vivo. (A, B)** ELISA analysis of IL-18 **(A)** and TNF-α **(B)** levels in bronchoalveolar lavage fluid (BALF) from SENP6*^fl/fl^* and SENP6*^fl/fl^* *Lyz2-cre* mice 12 h after intraperitoneal (i.p.) LPS injection (saline: n=3; LPS: n=6). **(C-E)** ELISA analysis of serum levels of IL-1β **(C)**, IL-18 **(D)**, and TNF-α **(E)** in SENP6*^fl/fl^* and SENP6*^fl/fl^* *Lyz2-cre* mice 12 h after i.p. injection of alum (700 μg) (saline: n=3; alum: n=6). **(F-H)** ELISA analysis of IL-1β **(F)**, IL-18 **(G)**, and TNF-α **(H)** levels in peritoneal lavage fluid (PLF) from SENP6*^fl/fl^* and SENP6*^fl/fl^* *Lyz2-cre* mice following alum administration (saline: n=3; alum: n=6). **(I, J)** Representative images of H&E-stained lung sections from SENP6*^fl/fl^* and SENP6*^fl/fl^* *Lyz2-cre* mice 12 h after i.p. alum injection. Scale bars: 100 μm. **(K)** Immunoblot analysis of lung lysates from SENP6*^fl/fl^* and SENP6*^fl/fl^* *Lyz2-cre* mice 24 h after LPS intratracheal instillation (n=6). **(L)** Lung W/D weight ratio in SENP6*^fl/fl^* and SENP6*^fl/fl^* *Lyz2-cre* mice after 24 h after LPS intratracheal instillation (2 mg/50 μL) (saline: n=3; LPS: n=6). **(M-O)** ELISA analysis of serum levels of IL-1β **(M)**, IL-18 **(N)**, and TNF-α **(O)** in SENP6*^fl/fl^* and SENP6*^fl/fl^* *Lyz2-cre* mice 12 h after 24 h after i.t. LPS treatment (saline: n=3; alum: n=6). **(P-R)** ELISA analysis of BALF levels of IL-1β **(P)**, IL-18 **(Q)**, and TNF-α **(R)** in SENP6*^fl/fl^* and SENP6*^fl/fl^* *Lyz2-cre* mice 24 h after i.t. LPS treatment (saline: n=3; alum: n=6). Data are presented as mean ± SD. Statistical analysis was performed using Student’s t-test (K) or Two-way ANOVA with Bonferroni test, based on n = 3 independent biological replicates for the saline group and n = 6 independent biological replicates for the LPS group. ***P* < 0.01, ****P* < 0.001.

**
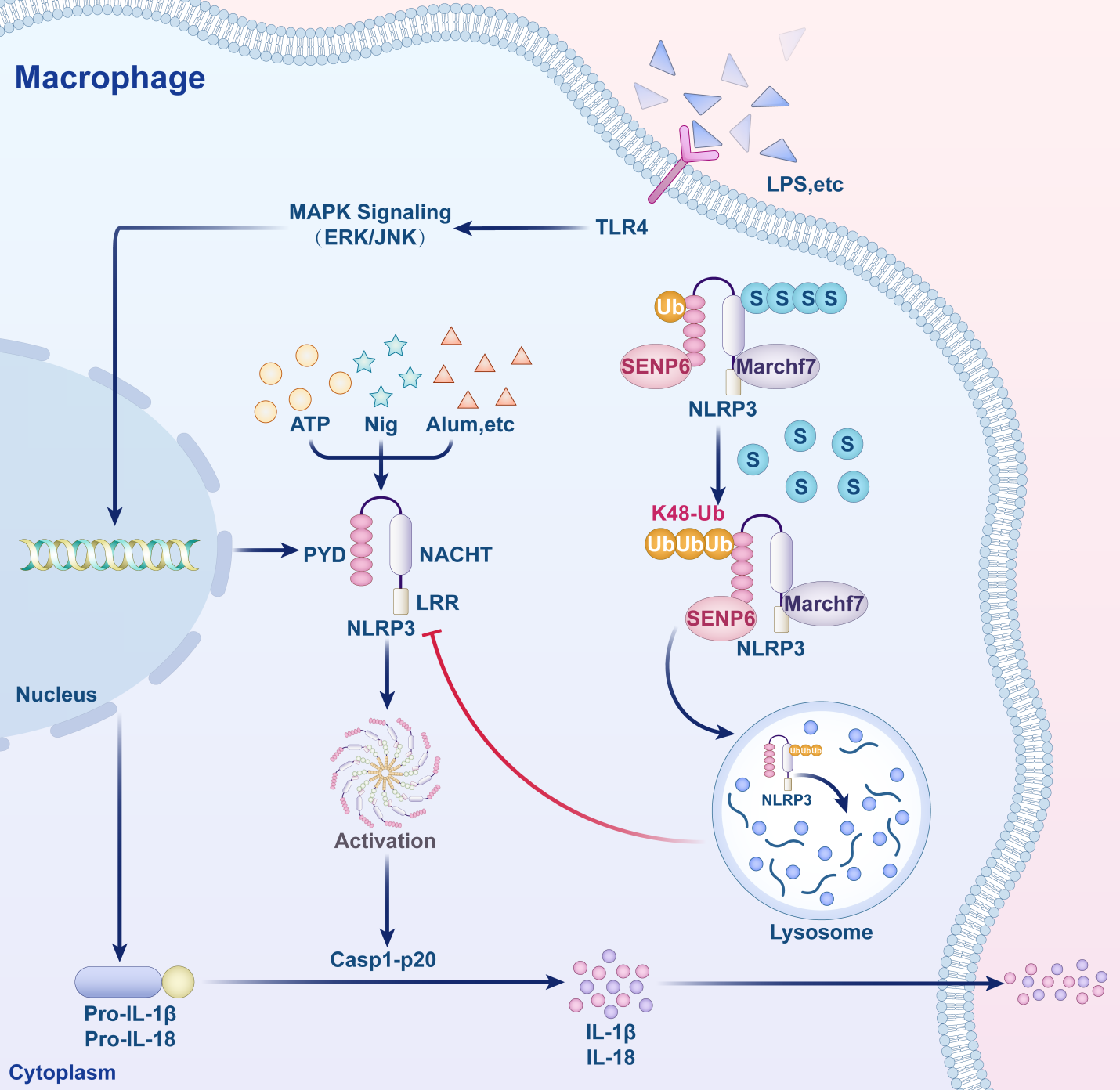
**

**Figure S6.** The schematic illustrating proposed mechanism of SENP6 regulation of NLRP3 inflammasome activation in macrophages.

**Supplementary Table S1**

**Antibodies used for Western blot/Immunoprecipitation.**

| **Antibody** | **Dilution ratio** | **Source** | **Identifer** |
| --- | --- | --- | --- |
| SENP6 | WB 1:1000  IP 1:500 | Sigma-Aldrich | QC22727 |
| NLRP3 | WB 1:1000  IP 1:400 | Cell signaling technology | 15101S |
| CASP1 | WB 1:1000 | Cell signaling technology | 3866S |
| IL-1β | WB 1:1000 | Cell signaling technology | 63124S |
| ASC | WB 1:1000 | Epizyme Biotech | R013826 |
| MARCHF7 | WB 1:100 | Santa Cruz Biotechnology | sc-166945 |
| β-actin | WB 1:50000 | Proteintech | 66009-1-Ig |
| NLRC4 | WB 1:1000 | Cell signaling technology | 12421S |
| AIM2 | WB 1:1000 | Cell signaling technology | 12948S |
| HA-tag | WB 1:6000  IP 1:50 | Proteintech | 51064-2-Ap |
| Myc-tag | WB 1:5000  IP 1:50 | Epizyme Biotech | LF-301S |
| DDDDK/Flag-tag | WB 1:1000  IP 1:50 | Proteintech | 66008-3-Ig |
| His-tag | WB 1:10000  IP 1:100 | Proteintech | 66005-1-Ig |
| SUMO1 | WB 1:1000 | Proteintech | 10329-1-Ap |
| SUMO2/3 | WB 1:1000 | Proteintech | 11251-1-Ap |
| p65 | WB 1:2000 | Proteintech | 10745-1-Ap |
| p-p65 | WB 1:2000 | Proteintech | 82335-1-RR |
| iκbα | WB 1:5000 | Proteintech | 10268-1-AP |
| p-iκbα | WB 1:1000 | Proteintech | 82349-1-RR |
| ERK1/2 | WB 1:2000 | Proteintech | 11257-1-AP |
| p-ERK1/2 | WB 1:1000 | Proteintech | 28733-1-AP |
| JNK | WB 1:2000 | Proteintech | 24164-1-AP |
| p-JNK | WB 1:1000 | Proteintech | 80024-1-RR |
| Anti-rabbit IgG HRP-linked Antibody | WB 1:10000 | ZSGB-bio | ZB-2301 |
| Anti-mouse IgG HRP-linked Antibody | WB 1:10000 | ZSGB-bio | ZB-2305 |
| Goat anti-rabbit Alexa Flour 488 | IF 1:400 | Invitrogen | WF322198 |
| Goat anti-mouse Alexa Flour 488 | IF 1:400 | Invitrogen | WF323912 |
| Goat anti-rabbit Alexa Flour 594 | IF 1:400 | Invitrogen | WK333741 |
| Donkey anti-mouse Alexa Flour 594 | IF 1:400 | Invitrogen | 2474956 |

**Supplementary Table S2**

**Primer sequences of certain genes used in qRT-PCR**

| **Gene name** | **Sequence** |
| --- | --- |
| ***mus-Nlrp3*** |  |
| Forward | 5′-TCTGCACCCGGACTGTAAAC-3′ |
| Reverse | 5′-CATTGTTGCCCAGGTTCAGC-3′ |
| ***homo-Nlrp3*** |  |
| Forward | 5′-CCACAACCCTCTGTCTACATTAC-3′ |
| Reverse | 5′-GCCCCATCTAACCCATGCTTC-3′ |
| ***mus-Senp6*** |  |
| Forward | 5′-ATGCAGACAAAGATGGGGCA-3′ |
| Reverse | 5′-CAGTCTTGCTCCGCCTTACA-3′ |
| ***mus-Marchf7*** |  |
| Forward | 5′-GGGTTGTTTCATCTCAAAGAC-3′ |
| Reverse | 5′-TCAAGGAATCCTGACTAGATC-3′ |
| ***mus-Il1b*** |  |
| Forward | 5′-GTGTCTTTCCCGTGGACCTT-3′ |
| Reverse | 5′-AATGGGAACGTCACACACCA-3′ |
| ***mus-Senp1*** |  |
| Forward | 5′-TGACCTTAGTGAACCACGGC-3′ |
| Reverse | 5′-AGGGTTATCTGAGTGATAATCTGG-3′ |
| ***mus-Senp2*** |  |
| Forward | 5′-GCCCTCCTTTGGTTTTACGC-3′ |
| Reverse | 5′-TTGCTTTTGCTATGGCGACG-3′ |
| ***mus-Senp3*** |  |
| Forward | 5′-CTCAAGTCCGGTGGTGGTTT-3′ |
| Reverse | 5′-GAGGCATCAAAAGATGGCCG-3′ |
| ***mus-Senp5*** |  |
| Forward | 5′-TGGAAGTCTGGTCCCACTCA-3′ |
| Reverse | 5′-ATGTCTGGCCCGGTAGTTTG-3′ |
| ***mus-Senp7*** |  |
| Forward | 5′-GTGGACAAGAAGCCTAAGAAATAAA-3′ |
| Reverse | 5′-TCCCTTAGGAGGACACCCAC-3′ |
| ***mus-β-actin*** |  |
| Forward | 5′-GGCTGTATTCCCCTCCATCG-3′ |
| Reverse | 5′-CCAGTTGGTAACAATGCCATGT-3′ |
| ***homo-β-actin*** |  |
| Forward | 5′-GTATCCTGACCCTGAAGTACC-3′ |
| Reverse | 5′-GAAGGTCTCAAACATGATCT-3′ |

### **Supplementary Table S3**

### The si-RNA sequences targeting indicated genes were listed

| **Gene** | **Sequence** |
| --- | --- |
| **Negative Control** |  |
| Forward | 5′-UUCUCCGAACGUGUCACGUTT-3′ |
| Reverse | 5′-ACGUGACACGUUCGGAGAATT-3′ |
| ***mus Senp6#1*** |  |
| Forward | 5′-GCUGACCGAAUUCAUAUAUTT-3′ |
| Reverse | 5′-AUAUAUGAAUUCGGUCAGCTT-3′ |
| ***mus Senp6#2*** |  |
| Forward | 5′-GCCUCAAAGAAGUGUUUGATT-3′ |
| Reverse | 5′-UCAAACACUUCUUUGAGGCTT-3′ |
| ***mus Senp6#3*** |  |
| Forward | 5′-GGGAGGCAUUUCUGUUACUTT-3′ |
| Reverse | 5′-AGUAACAGAAAUGCCUCCCT-3′ |
| ***mus Marchf7#1*** |  |
| Forward | 5′-GCACUCGUGUCCGAUUUAUTT-3′ |
| Reverse | 5′-AUAAAUCGGACACGAGUGCTT-3′ |
| ***mus Marchf7#2*** |  |
| Forward | 5′-CAGCAAUCUUAUGGAUUAUTT-3′ |
| Reverse | 5′-AUAAUCCAUAAGAUUGCUGTT-3′ |
| ***mus Marchf7#3*** |  |
| Forward | 5′-GCAGCGUCAUCAUCUAAUUTT-3′ |
| Reverse | 5′-AAUUAGAUGAUGACGCUGCTT-3′ |
| ***homo Senp6*** |  |
| Forward | 5′-GAUCCUGUAGAGAUUAUAUTT-3′ |
| Reverse | 5′-AUAUAAUCUCUACAGGAUCTT-3′ |
| ***homo Marchf7*** |  |
| Forward | 5′-GACUCCAGAAAUUCUUUAATT-3′ |
| Reverse | 5′-UUAAAGAAUUUCUGGAGUCTT-3′ |
